# The autonomic nervous system-lung interface in experimental BPD: NPY modulates immune response, alveolar growth and vascular muscularization in neonatal mice exposed to oxidative stress

**DOI:** 10.1186/s12931-026-03773-5

**Published:** 2026-07-01

**Authors:** Clemens O. Nies, Caroline Zeitouny, Celien Kuiper-Makris, Dharmesh Hirani, Jaco Selle, Christoph Bartz, Christina Vohlen, Jule Schmidt, Ruth Janoschek, Ivana Mižik, Margarete Odenthal, Silke v. Koningsbruggen-Rietschel, Herbert Herzog, Werner Seeger, Julian U.G. Wagner, Jörg Dötsch, Julian Koenig, Miguel A. Alejandre Alcazar

**Affiliations:** 1 Faculty of Medicine and University Hospital Cologne, Translational Experimental Pediatrics, Department of Pediatric and Adolescent Medicine, University of Cologne, Cologne, Germany; 2 Faculty of Medicine and University Hospital Cologne, Department of Child and Adolescent Psychiatry, Psychosomatics and Psychotherapy, University of Cologne, Cologne, Germany; 3 Faculty of Medicine and University Hospital Cologne, Department of Pediatric and Adolescent Medicine, University of Cologne, Cologne, Germany; 4 Institute for Lung Health (ILH), Cardiopulmonary Institute (CPI), Member of the German Center for Lung Research (DZL), University of Giessen and Marburg Lung Center (UGMLC), Giessen, Germany; 5 Department of Translational Pulmonology and Translational Lung Research Center Heidelberg, Member of the German Center for Lung Research (DZL), University Hospital Heidelberg, Heidelberg, Germany; 6 Faculty of Medicine and University Hospital Cologne, Institute of Pathology, University of Cologne, Cologne, Germany; 7 Faculty of Medicine, School of Clinical Medicine, University of New South Wales, Sydney, Australia; 8 St. Vincent’s Centre for Applied Medical Research, Darlinghurst, Sydney, NSW Australia; 9 Institute of Cardiovascular Regeneration, Centre for Molecular Medicine, Cardiopulmonary Institute (CPI), and German Center for Cardiovascular Research (DZHK), Partner Site Rhein-Main, Goethe University Frankfurt, Frankfurt a.M., Germany; 10 Faculty of Medicine and University Hospital Cologne, Cologne Excellence Cluster on Aging and Aging-Associated Diseases (CECAD), University of Cologne, Center for Molecular Medicine Cologne (CMMC), Cologne, Germany

**Keywords:** Hyperoxia, Bronchopulmonary dysplasia (BPD), Alveolar type 2 cells (AT2), Autonomic nervous system (ANS), Sympathetic nervous system (SNS), Neuropeptide Y (NPY)

## Abstract

Oxygen supplementation triggers inflammation and disrupts alveolar and microvascular growth in preterm infants, often leading to bronchopulmonary dysplasia (BPD). The autonomic nervous system (ANS) is critical in lung tissue homeostasis and repair. The sympathetic co-neurotransmitter neuropeptide Y (NPY) emerges as a central regulator of the ANS-organ interface with immune-modulatory function. Here, we studied sympathetic nervous system (SNS) signaling and the contribution of NPY to a hyperoxia-based model of BPD. To this end, neonatal wild-type (WT) and NPY knockout mice (NPY^−/−^) were exposed to 85% O_2_ (HYX) or 21% O_2_ (NOX) from birth to postnatal day 14. Prolonged hyperoxia caused a 7-fold increase of tyrosine hydroxylase (TH) protein, an enzyme characteristic for the SNS, and NPY mRNA (> 40-fold) in neonatal WT lungs. The analysis of lung scRNA-seq revealed an upregulation of NPY in alveolar macrophages of WT^HYX^ when compared to WT^NOX^. In contrast, NPY^−/− HYX^ showed lower amount of TH than WT^HYX^, indicating reduced sympathetic-associated signaling. Quantitative histomorphometry demonstrated an aggravated hyperoxia-induced alveolar growth arrest and septal thickening in NPY^−/− HYX^, whereas vascular muscularization and proliferation of vascular smooth muscle cells (SMC) were attenuated compared to WT^HYX^. Additionally, NPY^−/− HYX^ were protected from hyperoxia-induced CD68^+^ macrophage recruitment, despite exhibiting higher lung expression of *Il6* (20-fold) and *Il1b* (4-fold) than WT^HYX^. In summary, our data demonstrate a dual role of NPY in neonatal lungs in response to hyperoxia, preserving alveolarization while promoting immune cell recruitment and vascular muscularization, highlighting the importance of ANS-lung interface in lung maturation and injury.

**Supplementary Information:**

The online version contains supplementary material available at https://doi.org/10.1186/s12931-026-03773-5.

## Introduction

Preterm birth presents a significant global health challenge, as preterm infants face an increased risk of mortality, respiratory distress and long-term pulmonary complications [1, 2]. Due to the immaturity of their lungs, these infants often require respiratory support, including mechanical ventilation and/or oxygen supplementation. However, while these medical interventions are life-saving, their prolonged use interferes with normal lung morphogenic programming, thereby disrupting alveolar and microvascular growth and maturation. This defective alveolarization often leads to neonatal chronic lung disease (CLD), initially known as bronchopulmonary dysplasia (BPD) [3]. Inflammation is a key aspect of BPD pathogenesis [4], plays a central role in lung injury and is associated with the depletion of alveolar epithelial type 2 (AT2) cells, alveolar progenitors that are critical for alveolar growth, regeneration, and maintenance of alveolar integrity [5, 6].

The autonomic nervous system (ANS) is central in regulating key processes of lung development, including airway smooth muscle tone, vascularization, and alveolarization [7] .Through its intricate network of nerves, the ANS helps to coordinate lung function throughout life. Beyond its role in lung mechanics, the ANS has been shown to be a crucial regulator of immune response and tissue homeostasis, particularly in response to injury and inflammation [8, 9]. The ANS consists of two primary branches, the sympathetic nervous system (SNS) and the parasympathetic nervous system (PNS), which function antagonistically to maintain physiological homeostasis [10]. Interestingly, the SNS exerts its effects through the simultaneous release of norepinephrine (NE) and co-neurotransmitters such as neuropeptide Y (NPY). Together, they regulate lung function by influencing airway tone and pulmonary vascular dynamics [11, 12]. NPY is a highly conserved 36-amino acid peptide abundantly distributed in the central and peripheral nervous system where it modulates a wide range of physiological functions [12, 13]. Notable amongst the homeostatic regulatory processes are appetite regulation, anxiety and depression-related behaviors, nociception, and cardiorespiratory function [14]. Of note, NPY is shown to be implicated in various inflammatory lung pathologies. It has not only been associated with allergic airway inflammation, bronchial asthma, and lung fibrosis [15–18], but also with inflammation-related aberrant muscularization of pulmonary arteries in the context of pulmonary hypertension [11]. Highlighting its pleiotropic effects, NPY also influences multiple immune cell populations, exhibiting cytokine-like properties [19].

Despite the well-established function of NPY in tissue homeostasis and lung pathology, its impact on alveolar maturation and growth in the context of hyperoxia-based BPD remains elusive. Based on its contribution to inflammatory processes, immune cell regulation, and tissue injury, we now investigated the contribution of NPY to hyperoxia-induced neonatal lung injury using neonatal NPY knockout (NPY^−/−^) and wildtype (WT) mice. Specifically, we hypothesized that NPY deficiency is beneficial and would enable alveolarization and microvascular formation through reduced inflammation in response to an activated SNS. Interestingly, our analysis did not fully support this hypothesis but indicate a dual function of NPY in hyperoxia-induced neonatal lung injury. While the loss of NPY aggravates hyperoxia-induced arrest of alveolarization, it partly prevents vascular remodeling and recruitment of macrophages in hyperoxia-exposed neonatal mouse lungs. These findings indicate a cell- and compartment-specific function of NPY in alveolarization and neonatal lung injury, and thus suggesting that NPY cannot be categorized simply as “friend” (protective) or “foe” (pathogenic) in neonatal lung injury.

## Materials and methods

Detailed materials and methods are provided in the supplementary information.

### Animal experiments

Animal studies were conducted in compliance with local authorities (LANUV, NRW, Germany; AZ8.87-51.04. 2010.A372; 84-02.04. 2015.A120; AZ8.87-51.05.20.11.013), and studies were performed as previously described [5, 20]. Briefly, on postnatal day 0 (P0), female and male wildtype (WT) C57BL/6 and NPY-knockout B6.129 S-NPY^tm1Rpa/J^ pups (NPY^−/−^) were randomly assigned to experimental groups and exposed to 85% O_2_ (hyperoxia, HYX) in a Biospherix chamber and ProOx 110 Cytocentric^®^ High Infusion Rate O_2_ Controller. Control pups were maintained in room-air (normoxia, NOX, 21% O_2_). The dams were rotated daily between hyperoxia and normoxia to avoid adverse effects on maternal behavior or lactation. All mice were euthanized at P14, and lung tissues were collected for molecular and histological analyses. Detailed procedures are provided in the Supplementary Information.

## Tissue assays

### Protein extraction and Western Blot

Lungs were snap-frozen and stored at -80 °C for protein extraction and assessment of protein abundance using immunoblots, as previously described [20, 21]. Briefly, tissues were first homogenized, and protein concentrations were determined prior to analysis. Protein abundance was then evaluated by immunoblotting, as previously described [5]. Detailed information of the antibodies is provided in the Supplementary Information.

### RNA extraction, cDNA synthesis, and quantitative reverse transcriptase polymerase chain reaction PCR (qRT-PCR)

Total RNA was extracted from lungs using a previously described protocol [5], and complementary DNA (cDNA) was synthesized using reverse transcription. The qRT-PCR was then performed and mRNA expression was related to *Polr2a, Gapdh, or Actb* serving as housekeeping genes. A list of primer sequences is provided in Supplementary Table 1.

### Single cell RNA sequencing (scRNA-seq) data

Publicly available scRNA-seq dataset from newborn mice were re-analyzed as previously described [22] to assess the number of *Npy*⁺ cells within the alveolar macrophage population and NPY gene expression in the developing mouse lungs at single-cell level. Details on proportion of *Npy*^+^ cells in lung myeloid cells are summarized in Table 2 (provided as an Excel file, Supplementary Table 2). Proportion of *Npy*^+^ cells in alveolar macrophages cluster was analyzed with GrapPad Prism 10.2.1.

### Quantitative histomorphometric, immunohistochemical and immunofluorescent analysis

PFA-fixed lungs were embedded in paraffin for isotropic uniform random (IUR) sectioning, followed by histomorphometric analysis, immunohistochemical staining or immunofluorescent staining, as described previously [5, 15]. Briefly, PFA-fixed, paraffin-embedded lungs were sectioned at 3 μm, mounted on poly-L-lysine-coated slides, deparaffinized in NeoClear (Merck, Germany, #1098435000), and rehydrated through graded ethanol.

For histomorphometric analysis, sections were stained with hematoxylin (Carl Roth, Germany, #T865.2) and 0.5% eosin (Carl Roth, Germany, #X883.2), dehydrated, cleared, and mounted. Mean linear intercept (MLI), radial alveolar count (RAC), and alveolar septal thickness (AST) were quantified by light microscopy using a Leica SCN400 slide scanner and Cell D 3.4 software (Olympus), analyzing 4–5 sections from 5 animals per group and up to 10 images per section.

For extracellular matrix analysis, elastic fibers were stained with 10% Resorcin-Fuchsin according to Weigert (Waldeck Chroma, Germany, #2.00E-30) in 1% hydrochloric acid/70% ethanol overnight and counterstained with 0.5% tartrazine (ScyTek Laboratories, USA, #TZQ125) in 0.25% acetic acid for 30 min. Collagen fibers were stained with Picrosirius Red (ScyTek Laboratories, USA, #SRS500) and counterstained with 0.2% phosphomolybdic acid (Carl Roth, Germany, #4440.1). Elastic and collagen fiber density were quantified relative to total lung tissue area using ImageJ.

For immunohistochemistry, antigen retrieval was performed in 10 mM citrate buffer (pH 6, Dako, Germany, #S2369) followed by blocking and incubation with rabbit anti-CD68 (Abcam, UK, #ab125212, 1:200) overnight at 4 °C. Detection was performed using the Histofine^®^ MOUSESTAIN KIT (Nichirei, Japan, #414341F). CD68^+^ cells and medial wall thickness (MWT) were assessed in transversally cut vessels < 20 μm and 20–100 μm, normalized to lung area.

For immunofluorescence staining, sections were processed for antigen retrieval and blocking as described above. Proliferating vascular smooth muscle cells (SMC) were identified using Ki-67 (Invitrogen, USA, #14-5698-82, 1:500) and αSMA-Cy3 (Sigma-Aldrich, USA, #C6198, 1:200), followed by DAPI (Sigma-Aldrich, USA, #D9542, 1:1000). Ki-67^+^/αSMA^+^ cells were quantified in vessels of 20–100 μm diameter and normalized to total αSMA^+^ cells. For sympathetic innervation, sections were stained with rabbit anti-TH (Abcam, UK, #ab112, 1:200) followed by secondary goat anti-rabbit AF488 antibody (Jackson ImmunoResearch, 1:200); TH^+^ signal was quantified relative to DAPI^+^ nuclei. For combined TH/NPY detection, sections were incubated with rabbit anti-TH (Abcam, UK, #ab112, 1:200) and rabbit anti-NPY (Cell Signaling Technology, USA, #11976, 1:200), followed by AF488- and Cy3-conjugated secondary antibodies (Jackson ImmunoResearch, 1:200). Images were acquired on an Olympus BX43 microscope and analyzed using CellSens Dimension software; representative images were obtained using a Stellaris 5 confocal microscope (Leica Microsystems).

### Statistical analysis

All data are presented as mean ± SEM. Sample sizes ranged from *n* = 4–12 animals per group, as indicated in the respective figure legends. Statistical analyses were performed using non-parametric Mann-Whitney tests or two-way ANOVA followed by Bonferroni post hoc testing, as appropriate.

## Results

### Hyperoxia increases abundance of neuronal markers, tyrosine hydroxylase (TH), and expression of *Npy* in neonatal mouse lungs

**Fig. 1:**
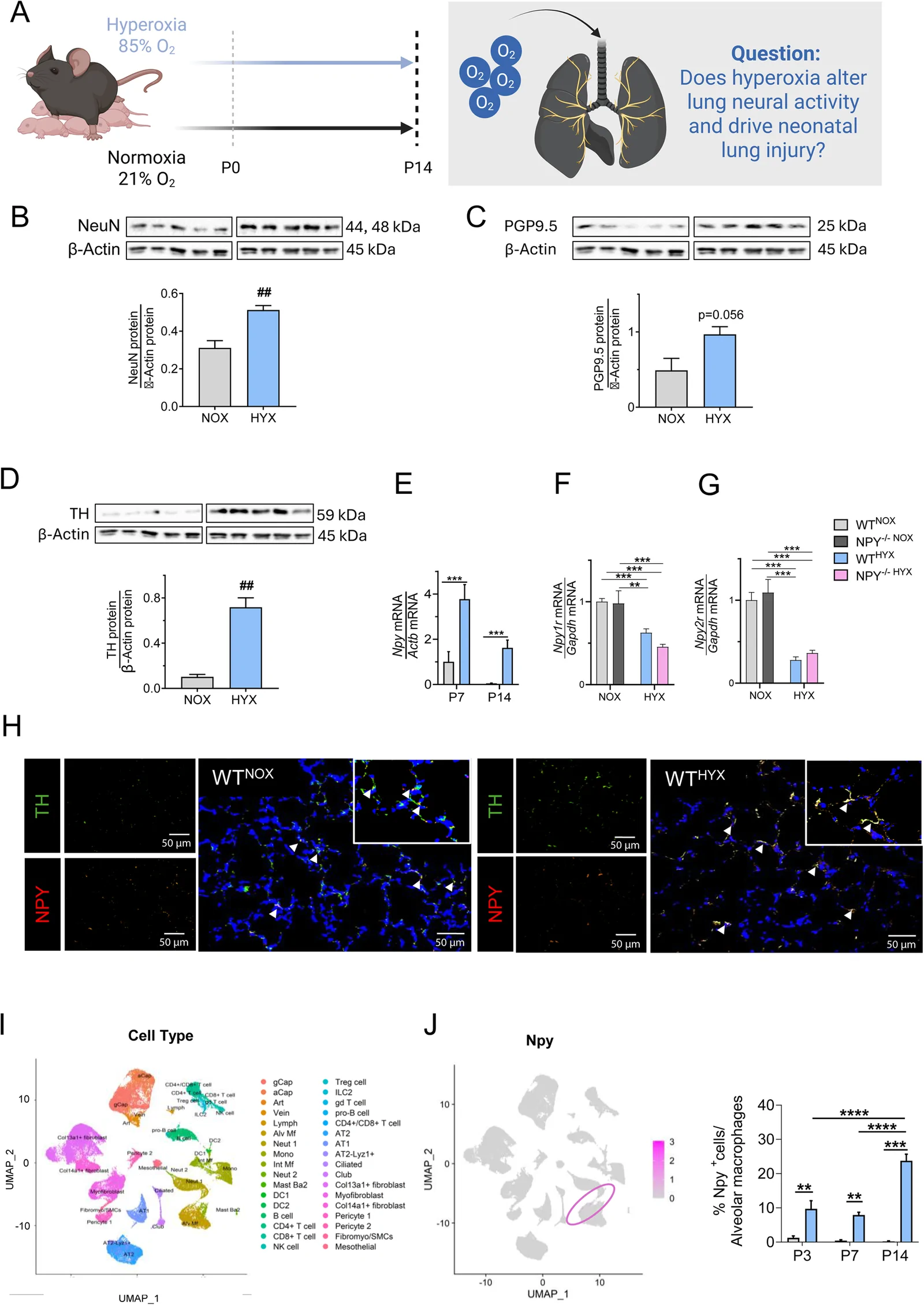
**A** Scheme illustrating the research question and the experimental model of hyperoxia-based bronchopulmonary dysplasia (BPD) in which neonatal wildtype mice are exposed to 85% O_2_ (hyperoxia, HYX) or 21% O_2_ (normoxia, NOX) from birth (postnatal day 0, P0) to P14. **B**–**D** Immunoblots showing NeuN (neuronal marker; **B**), Protein Gene Product 9.5 (PGP9.5, a marker for nerve fibers; **C**), and Tyrosine Hydroxylase (TH, a marker of the sympathetic nerve fibers; **D**) in total lung homogenate at P14; total abundance was related to β-Actin that served as loading control. The densitometric summary data are shown under the respective immunoblot. **E** Gene expression of *Npy* using quantitative RT-PCR; Actin b (*Actb*) served as housekeeping gene. **F**, **G** Gene expression of *Npy1r* (**F**) and *Npy2r *(**G**) using quantitative RT-PCR; *Gapdh *served as housekeeping gene. **H** Representative immunofluorescent images showing staining for tyrosine hydroxylase (TH, green) and NPY (red) in lung sections of newborn wild-type (WT^HYX^) and NPY knockout (NPY^⁻/⁻ HYX^) mice exposed to HYX from birth (P0) to P14. Co-localization of TH and NPY signals (yellow in merged images) is indicated by arrows. **I** UMAP plot depicting cell clusters identified in lungs from 3, 7 or 14 days-old, NOX or HYX-exposed mouse pups. Data are a re-presentation of the original article by Hurskainen, Mizikova et al. (Nat Commun, 2021) and is reproduced under the creative commons barbiton 4.0 international license (https://creativecommons.org/licenses/by/4.0/). **J** Feature plot indicating *Npy* expression across all lung cell clusters as indicated in (**I**). Bar graph depicting the percentual contribution of *Npy*^+^ cells within the subpopulation of alveolar macrophages at P3, P7, and P14. All data are presented as mean ± SEM. Sample sizes ranged from *n* = 6–12 animals per group. Statistical significance was assessed using non-parametric Mann-Whitney test: ^##^*p* < 0.01; Two-way ANOVA with Bonferroni post-test: **p < 0.01, ***p < 0.001, ****p < 0.0001 unless otherwise indicated. Created in BioRender. Alejandre Alcazar, M (2026) https://BioRender.com/redldah

To investigate the impact of hyperoxia on pulmonary innervation (Fig. 1A), we first measured protein abundance of NeuN, a neuronal nuclear protein, often used as a marker to identify neurons. Prolonged exposure to postnatal hyperoxia caused a marked increase of NeuN protein when compared to normoxia-exposed WT pups at P14 (Fig. 1B), suggesting increased intrinsic neuronal network in the lung. A similar increase (*p* = 0.056) was found for pulmonary Protein Gene Product 9.5 (PGP9.5), a protein primarily found in nerve cells and used as a marker for nerve fibers (Fig. 1C). Tyrosine hydroxylase (TH) is a key enzyme in catecholamine synthesis and a marker of sympathetic nerve fibers [23]. Since strong evidence indicates that the SNS regulates immune cell function and triggers inflammation, we next assessed TH in lung homogenates from WT pups at P14. Hyperoxia resulted in significantly elevated TH protein concentrations in total lung homogenates when compared to normoxia-exposed control pups (Fig. 1D). To further strengthen the notion of a heightened sympathetic tone, we next measured NPY mRNA at P7 and P14 and detected significantly increased *Npy* expression in lungs of hyperoxia-exposed pups at both time points (Fig. 1E), supporting a sustained elevation in pulmonary sympathetic tone. To further characterize NPY signaling in the lungs, we assessed the expression of NPY receptors (*Npy1r* and *Npy2r*) and found significantly reduced mRNA expression of both *Npy1r* and *Npy2r* in both WT^HYX^ and NPY^−/− HYX^ when compared to WT^NOX^ and NPY^−/− NOX^, respectively (Fig. 1F, G). However, no marked differences in NPY receptor mRNA expression were observed between WT and NPY^−/−^ lungs under either condition. This finding indicates that NPY deficiency does not alter its receptor expression in the lung. To further investigate if NPY expression is associated with sympathetic innervation in the lung, we performed immunofluorescence staining for NPY and TH of a total of 4 animals per group. The representative images demonstrate a co-localization of NPY and TH signals in lung tissue (Fig. 1H), suggesting that a subset of NPY-expressing structures in the lungs is associated with sympathetic innervation. Moreover, we re-analyzed previously published single-cell RNA sequencing (scRNA-seq) data of lung cells from mice exposed to normoxic or hyperoxic conditions [22]. UMAP clustering of the SE dataset identified distinct lung cell populations, including alveolar macrophages, epithelial, endothelial, and mesenchymal cells, each defined by their transcriptional profiles. We found that NPY expression was increased in cells from hyperoxia-exposed lungs specifically within the alveolar macrophage population compared to those from normoxic controls, as visualized by UMAP expression plots. This increase was evident as early as P3 and progressed through P7 to P14, with over a 10-fold increase of *Npy*-expressing alveolar macrophages at P14 compared to normoxic controls (Fig. 1I, J). Collectively, these findings suggest that prolonged exposure to hyperoxia increases neuronal presence in the lungs, along with markers consistent with enhanced sympathetic signaling and expression of its co-neurotransmitter NPY, also in alveolar macrophages.

### NPY^−/−^ mice exhibit an aggravated hyperoxia-induced alveolar growth arrest

**Fig. 2:**
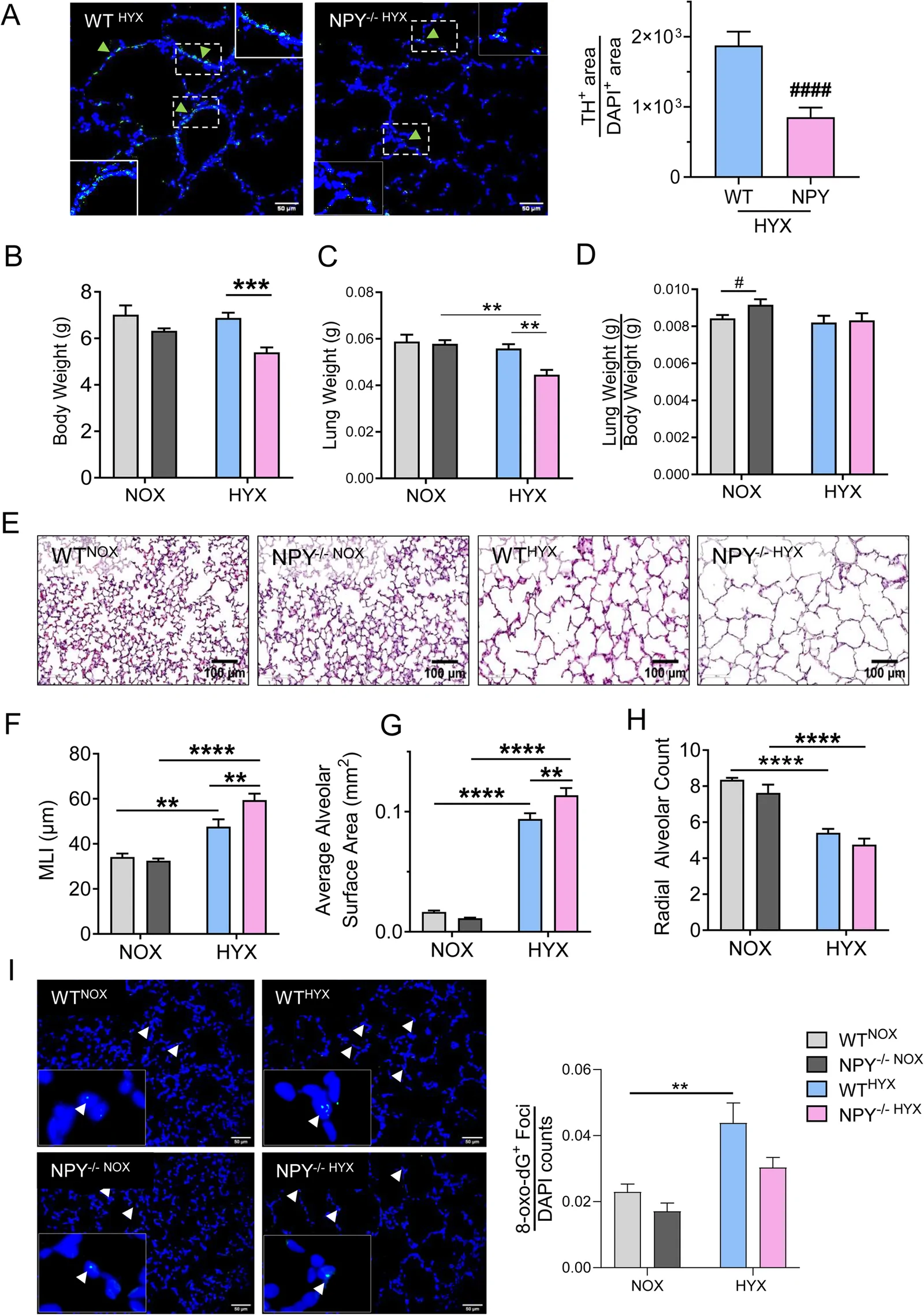
**A** Representative immunofluorescent images showing positive staining for Tyrosine Hydroxylase (TH, green arrows) in lungs of newborn wildtype (WT^HYX^) and NPY knockout mice (NPY^−/− HYX^) exposed to 85% O_2_ (hyperoxia, HYX) from birth (postnatal day 0, P0) to P14. Quantification of the TH^+^ area relative to DAPI^+^ area is shown next to the immunofluorescent images. **B** Body weight (g) of WT and NPY^−/−^ mice exposed HYX or 21% O_2_ (normoxia, NOX) from birth to P14. **C**, **D** Lung weight (g) (**C**) and Lung-Body weight ratio (**D**) of WT and NPY^−/−^ mice exposed HYX or NOX from birth to P14. **E** Representative images of hematoxylin & eosin-stained lung sections from WT^NOX^, NPY^−/− NOX^, WT^HYX^, and NPY^−/− HYX^. **F–H** Quantitative histomorphometry of lungs of mice exposed to HYX or NOX from birth until P14 using three parameters: mean linear intercept (MLI, µm) (**F**), average alveolar surface of a single alveolus per mm2 (**G**), and radial alveolar count (RAC) (**H**). **I** Representative immunofluorescent images showing positive staining for 8-hydroxy-2’- deoxyguanosine (8-oxo-dG, green) and DAPI (blue) in lungs of newborn WT and NPY^−/−^ exposed to HYX or NOX from birth P0 to P14. Quantification of the 8-oxo-dG positive foci relative to DAPI^+^ area is shown next to the immunofluorescent images. All data are presented as mean ± SEM. Sample sizes ranged from *n* = 4–6 animals per group and 4–8 lung sections per animal were analyzed. Statistical significance was assessed using non-parametric Mann-Whitney test or Two-way ANOVA with Bonferroni post-test: ^#^*p* < 0.05, **p < 0.01, ***p < 0.001, ****p < 0.0001

We next performed immunofluorescent staining for TH in lung sections of WT and NPY^−/−^ mice exposed to hyperoxia. The analysis revealed a ubiquitous reduction of TH in lungs of NPY^−/−^ mice after hyperoxia at P14, indicating that NPY alters sympathetic innervation in the lung under hyperoxic conditions (Fig. 2A). Since NPY^−/−^ mice showed a mitigated hyperoxia-induced sympathetic innervation (TH^+^ staining), we next investigated the effects of NPY deficiency on neonatal lung development. Interestingly, NPY^−/−^ mice exhibited a marked reduction in body weight after hyperoxia, an effect that was not observed in WT mice (Fig. 2B). Additional measurements showed that NPY^−/− HYX^ had lower lung weights than WT^HYX^ mice. However, when lung weight was normalized to body weight, no differences between the groups were detectable (Fig. 2C, D). To further examine lung structural changes, quantitative histomorphometric analysis was performed on H&E-stained isotropic uniform random (IUR) lung sections (Fig. 2E; Supplementary Fig. 1), and confirmed an impaired alveolar formation in WT^HYX^ mice compared to WT^NOX^, an effect further exacerbated and most pronounced in NPY^−/− HYX^ animals (Fig. 2E-H). Specifically, WT^HYX^ pups showed an increased mean linear intercept (MLI) (Fig. 2F), enlarged average alveolar size (Fig. 2G), and reduced radial alveolar count (RAC) (Fig. 2H), relative to WT^NOX^. While alveolar structure in NPY^−/− NOX^ mice was not significantly different from WT^NOX^, NPY^−/− HYX^ mice displayed more pronounced alveolar growth arrest, characterized by increased MLI and enlarged average alveolar size compared to both NPY^NOX^ and WT^HYX^ (Fig. 2E-H). To investigate whether oxidative stress could contribute to these changes, we next assessed oxidative-stress associated DNA damage in the lungs using 8-hydroxy-2’- deoxyguanosine (8-oxo-dG) staining, a well-established marker of ROS-induced oxidative DNA damage. The 8-oxo-dG-positive foci were increased in lungs of WT^HYX^, indicating an increased oxidative DNA damage following hyperoxia. Interestingly, NPY^−/− HYX^ mice exhibited an attenuated increase of 8-oxo-dG compared to WT^HYX^ (Fig. 2I). These findings indicate that loss of NPY exacerbates hyperoxia-induced arrest of alveolarization, suggesting an important role for NPY - and potentially the SNS - in alveolar growth and repair in neonatal mice exposed to hyperoxia.

### Hyperoxia-induced arrest of vascular formation is largely independent of NPY

**Fig. 3:**
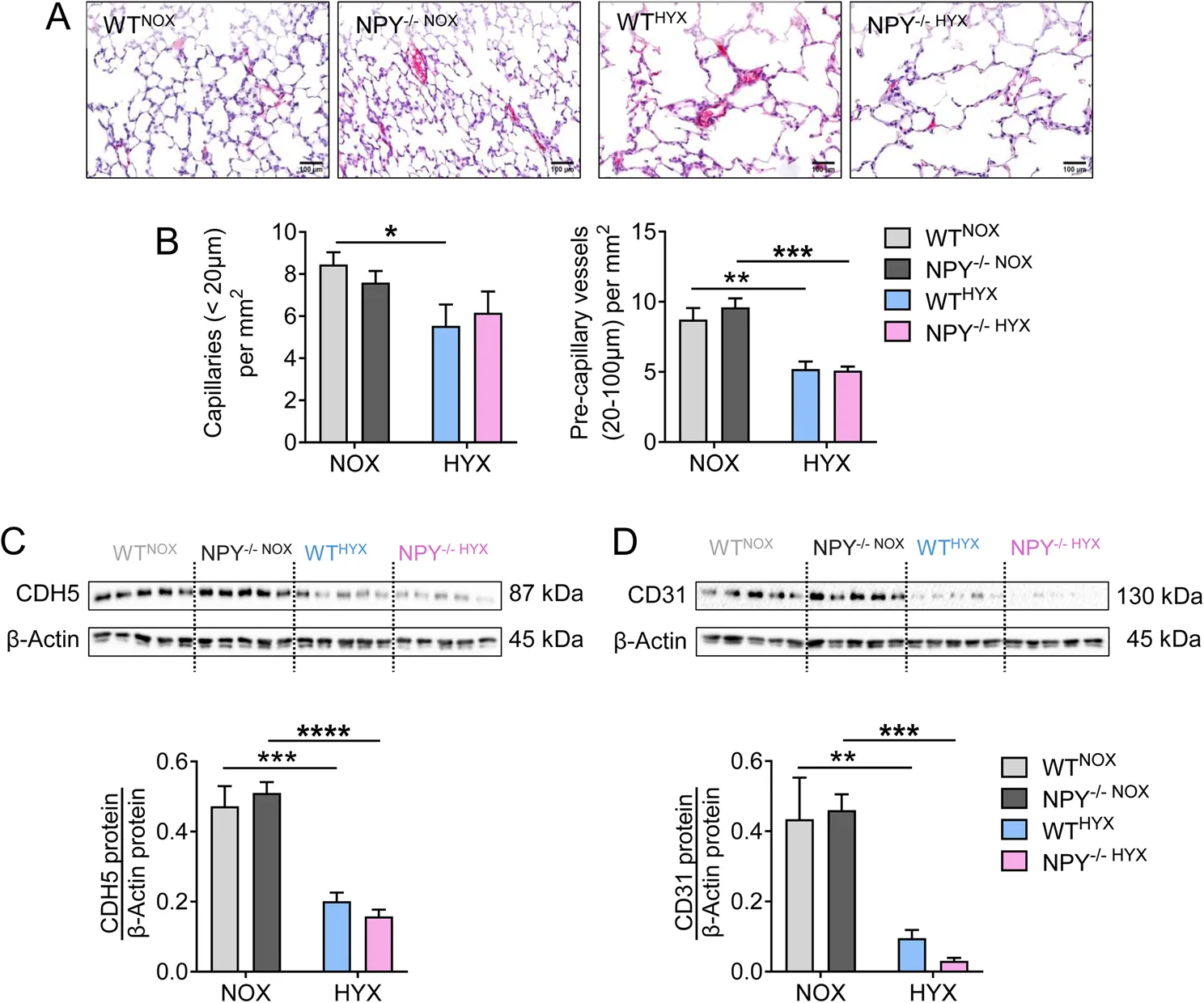
**A**, **B** Representative images showing dual staining of Willebrand Factor (vWF; endothelial marker) and alpha SMA (αSMA; marker of smooth muscle cells) in lungs of newborn wildtype (WT) and NPY knockout mice (NPY^−/−^) exposed to 21% O_2_ (normoxia, NOX) or 85% O_2_ (hyperoxia, HYX) from birth (postnatal day 0, P0) to P14. Quantification of capillaries (< 20 μm) and precapillary vessels (20–100 μm) (*n* = 4–6 animals per group and 4–8 lung sections per animal were analyzed). **C**, **D** Immunoblots showing vascular endothelial cadherin (VE-Cdh., CDH5) and PECAM (CD31) both endothelial markers in total lung homogenate at P14; total abundance was related to β-Actin that served as loading control (n = 6–12 animals per group). The densitometric summary data are shown under the respective immunoblot. All data are presented as mean ± SEM. Statistical significance was assessed using Two-way ANOVA with Bonferroni post-test: *p < 0.05, **p < 0.01, ***p < 0.001, ****p < 0.0001

Given the exacerbation of hyperoxia-induced alveolar growth arrest observed in NPY^-/-^ mice, we next examined if these structural deficits were associated with vascular alterations and dysregulated expression of genes related to endothelial cell function. First, lung sections were co-stained for von Willebrand factor (vWF) and αSMA at P14, and the formation of pre-capillary vessels was analyzed (Fig. 3A). For quantification of microvascular formation per defined lung tissue area (in millimeter), we differentiated between capillaries (< 20 μm in diameter) and pre-capillary vessels (20 to 100 μm in diameter). In WT mice, hyperoxia significantly reduced both the number of capillaries and pre-capillary vessels per mm^2^. On the contrary, capillaries were not significantly diminished in NPY^−/− HYX^ when compared to NPY^−/− NOX^, indicating that NPY deficiency partially preserves capillary density and capillary loss. However, no protective effect on pre-capillary vessel formation was observed in NPY^−/−HYX^ (Fig. 3B). These data show that NPY^HYX^ partially mitigated the reduction in capillary formation in neonatal lungs exposed to hyperoxia, but did not exhibit any protective effect on pre-capillary vessel number. Based on the preceding findings, we next examined the protein abundance of key endothelial markers, VE-cadherin (CDH5) and PECAM-1 (CD31) in lung homogenates. Hyperoxia caused a reduction of both proteins by more than 50% in WT and NPY^−/−^ pups (Fig. 3C, D). Since total lung homogenates were analyzed, Western blot assessment of endothelial cell markers reflects all vascular compartments. Consequently, subtle alterations within the capillary bed may not be sufficiently resolved, whereas changes in pre-capillary and larger vessels are more detected.

### NPY^−/−^ mice are partially protected from hyperoxia-induced lung vascular muscularization and vascular smooth muscle cell (SMC) proliferation

**Fig. 4:**
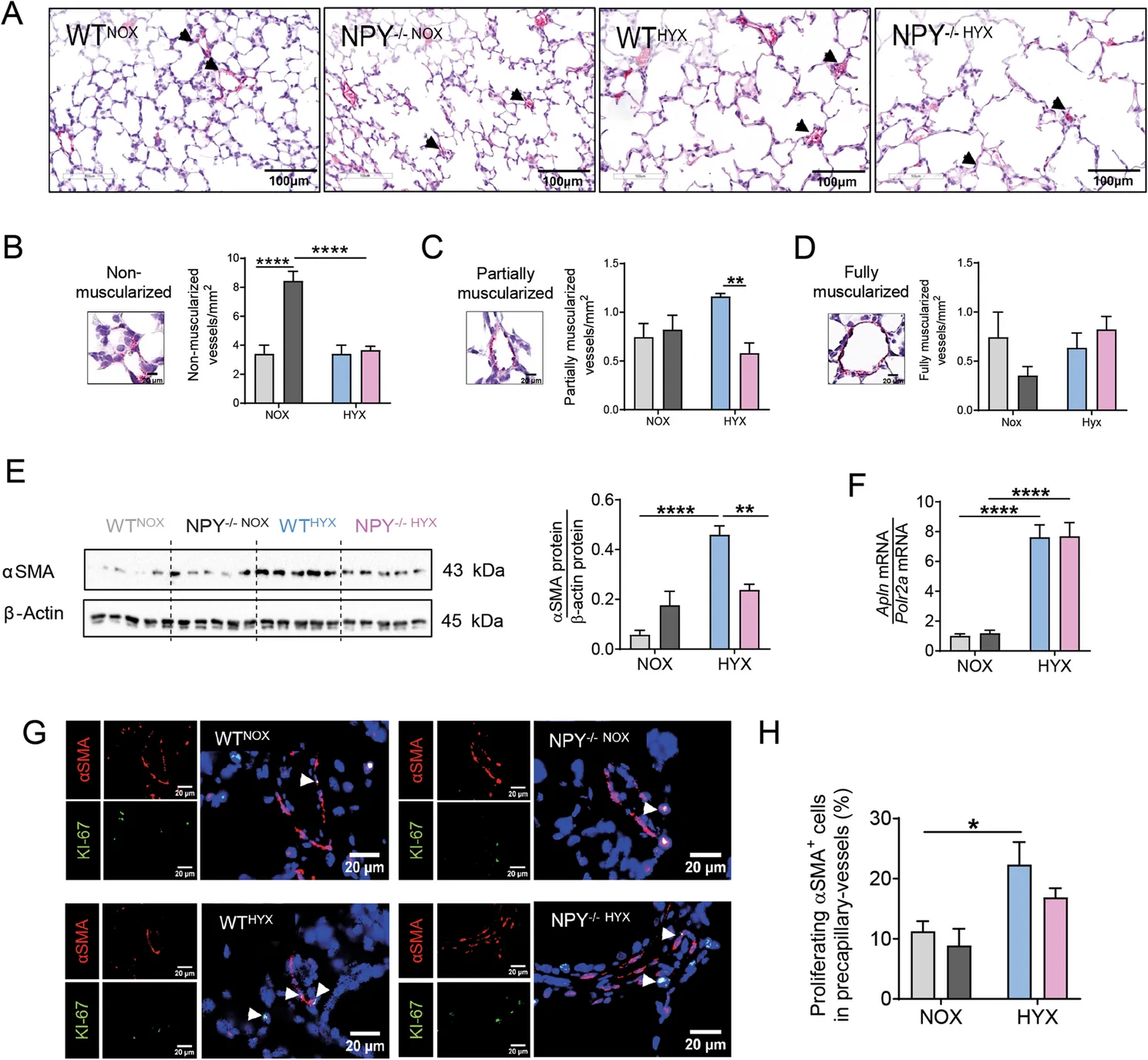
**A–D** Representative images showing dual staining of Willebrand Factor (vWF; endothelial marker) and alpha SMA (αSMA; marker of smooth muscle cells) in lungs of newborn wildtype (WT) and NPY knockout mice (NPY^−/−^) exposed to 21% O_2_ (normoxia, NOX) or 85% O_2_ (hyperoxia, HYX) from birth (postnatal day 0, P0) to P14 (**A**). Quantification of non-muscularized (**B**), partially muscularized (**C**), and fully muscularized pre-capillary (**D**) vessels per mm^2^ (*n* = 4–6 animals per group). **E** Immunoblot showing αSMA in total lung homogenate at P14; total abundance was related to β-Actin that served as loading control. The densitometric summary data are shown next to immunoblot. **F** Gene expression of apelin (*Apln*) using quantitative RT-PCR; *Polr2a* served as housekeeping gene (*n* = 6–12 animals per group). **G**, **H** Representative immunofluorescent staining for KI67 (proliferative marker) and αSMA in lungs at P14 (**G**). Quantitative data show KI67^+^ (proliferating) αSMA^+^ cells in precapillary vessels (< 100 μm) in % of all αSMA^+^ cells (*n* = 4–6 animals per group and 4–8 lung sections per animal were analyzed) (**H**). All data are presented as mean ± SEM. Statistical significance was assessed using Two-way ANOVA with Bonferroni post-test: *p < 0.05, **p < 0.01, ****p < 0.0001

Reports indicate a functional role of NPY in the homeostasis of SMC and the pathogenesis of pulmonary hypertension (PH) [11]. Therefore, we now investigated vascular muscularization in both WT and NPY^−/−^ mice after postnatal hyperoxia (Fig. 4A-D). First, under physiological normoxic conditions, we found that NPY^−/−^ pups exhibited a significantly higher density of non-muscularized pre-capillary lung vessels per mm² compared to WT controls. Hyperoxia, however, caused a marked reduction in the density of non-muscularized pre-capillary lung vessels per mm² in NPY^−/− HYX^ pups compared to NPY^−/− NOX^ (Fig. 4B). In contrast, the density of partially muscularized vessels showed no change in WT^NOX^ and NPY^−/− NOX^ pups. Under hyperoxic conditions, we found that the proportion of partially muscularized vessels in WT^HYX^ were slightly increased, whereas NPY^−/− HYX^ mice were fully protected from this effect (Fig. 4C). Moreover, the proportion of fully muscularized lung vessels was reduced in NPY^−/− NOX^ animals by trend, whereas hyperoxia slightly, but not significantly, increased the proportion of fully muscularized lung vessels in NPY^−/− HYX^ pups when compared to NPY^−/− NOX^ (Fig. 4D). These findings suggest that hyperoxia promotes an increase of partially muscularized vessels, whereas deficiency of NPY mitigates vascular remodeling and partial muscularization. To further support the notion of an NPY-mediated increase of perivascular ACTA2^+^ cells through proliferation, we first analyzed αSMA (ACTA2) protein abundance using immunoblotting. In WT^HYX^ compared to WT^NOX^ mice, the αSMA protein abundance was significantly increased, whereas this effect was significantly attenuated in NPY^−/− HYX^ (Fig. 4E). Moreover, the expression of apelin (*Apln*) was also significantly upregulated in both WT and NPY^−/−^ after hyperoxia (Fig. 4F). Consistently, hyperoxia induced a significant increase of proliferating vascular ACTA2^+^ cells (20–100 μm in diameter) in WT pups, whereas this effect was mitigated in NPY^−/−^ (Fig. 4G, H). Collectively, these data suggest that NPY partially contributes to the proliferation of perivascular ACTA2^+^ cells with vascular muscularization.

### NPY^−/−^ mice are protected against hyperoxia-induced pulmonary macrophage infiltration, but not against cytokine upregulation in neonatal mice

**Fig. 5:**
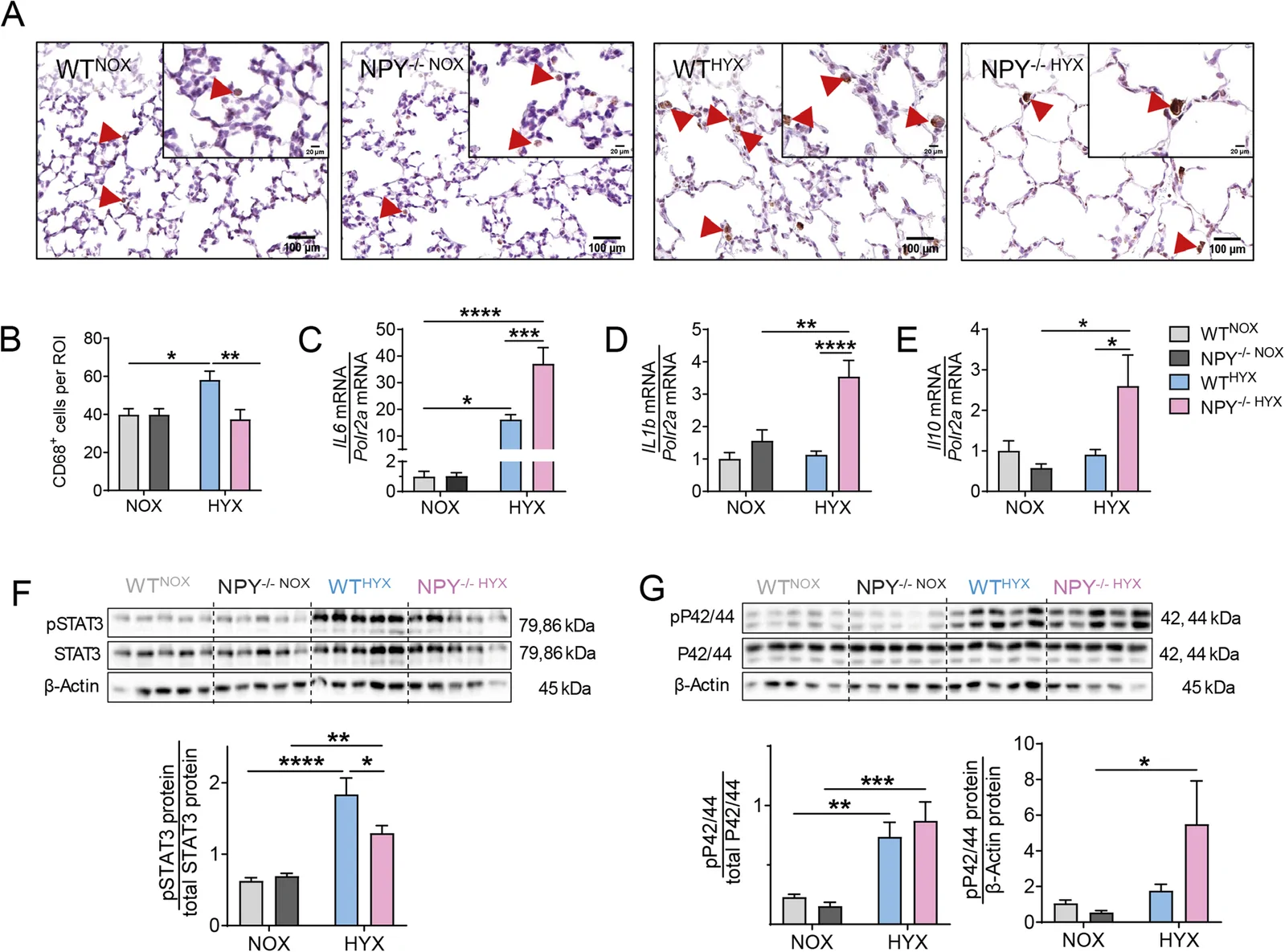
**A**, **B** Representative images showing staining of CD68 (macrophage marker) in lungs of newborn wildtype (WT) and NPY knockout mice (NPY^−/−^) exposed to 21% O_2_ (normoxia, NOX) or 85% O_2_ (hyperoxia, HYX) from birth (postnatal day 0, P0) to P14 (**A**). Quantification of CD68^+^ cells per region of interest (ROI) (**B**) in lungs of WT^NOX^, NPY^−/− NOX^, WT^HYX^, and NPY^−/− HYX^ (*n* = 4–6 animals per group and 4–8 lung sections per animal were analyzed). **C**–**E** Gene expression of interleukin 6 (*Il6*) (**C**), interleukin 1 beta (*Il1b*) (**D**), and interleukin 10 (*Il10*) (**E**) using quantitative RT-PCR; *Polr2a* served as housekeeping gene. **F** Immunoblot showing phosphorylated (*p*)STAT3 and total STAT3 in total lung homogenate at P14; pSTAT3 was related to total STAT3; β-Actin served as loading control (*n* = 6–12 animals per group). The densitometric summary data are shown under the respective immunoblot. **G** Immunoblot showing phosphorylated (*p*)P42/44 and total P42/44 in total lung homogenate at P14; pP42/44 was related to total P42/44 or to β-Actin that served as loading control (*n* = 6–12 animals per group). The densitometric summary data are shown under the respective immunoblot. All data are presented as mean ± SEM. Statistical significance was assessed using Two-way ANOVA with Bonferroni post-test: *p < 0.05, **p < 0.01, ***p < 0.001, *p < 0.0001

Inflammation is a key driver of BPD, contributing to lung injury and impaired alveolar development. Given that the SNS and its co-neurotransmitter NPY regulate immune response, we investigated the impact of NPY deficiency on inflammatory responses under hyperoxic conditions. To this end, we first assessed the recruitment of macrophages to the lung in WT vs. NPY^−/−^ mice exposed to normoxia or hyperoxia using immunohistochemical staining of lung sections for CD68, a marker for activated macrophages (Fig. 5A). In WT mice, hyperoxia significantly increased the number of CD68^+^ cells per field of view (Region of Interest, ROI). In contrast, this hyperoxia-mediated chemoattractant effect was abolished in NPY^−/− HYX^ compared to WT^HYX^ or NPY^−/− NOX^ (Fig. 5B). Next, we assessed the expression of genes encoding for key inflammatory cytokines and found a marked 20-fold induction of *Il6* expression, but no effects on *Il1b* and *Il10* expression in WT^HYX^ compared to WT^NOX^. On the other hand, NPY deficiency had a striking additive effect and potentiated the hyperoxia-induced inflammatory effect. Specifically, NPY^−/− HYX^ exhibited a 40-fold increased *Il6* expression compared to NPY^−/− NOX^, which is twice as high as the response observed in WT pups exposed to hyperoxia (Fig. 5C). Similarly, the gene expression of *Il1b* and *Il10* were 2-fold higher in NPY^−/− HYX^ compared to either NPY^−/− NOX^ or WT^HYX^ (Fig. 5D, E). Given the accumulating evidence of IL-6 being central in the pathomechanism of the arrest of alveolarization and the marked upregulation of *Il6* in NPY^−/−^ mice exposed to hyperoxia, we next investigated the activation of the IL-6 downstream effector, Signal Transducer and Activator of Transcription 3 (STAT3). However, despite the increased *Il6* in NPY^−/− HYX^, phosphorylation of STAT3 relative to total STAT3 was significantly mitigated compared to WT^HYX^ (Fig. 5F). To further assess inflammatory and regenerative signaling, we analyzed phosphorylation of Extracellular Signal-Regulated Kinases 1/2 (ERK1/2; P42/44). In contrast to STAT3 signaling, hyperoxia significantly increased P42/44 phosphorylation relative to the total P42/44 in both WT and NPY^−/−^ mice to the same extent. Interestingly, when P42/44 was related to the loading control (β-Actin), phosphorylated ERK1/2 abundance was significantly higher in NPY^−/− HYX^ compared to WT^NOX^ and NPY^−/− NOX^ (Fig. 5G). In summary, our data demonstrate a dual mechanism of NPY: on one hand NPY seems to favor the recruitment of macrophages to the lung; on the other it balances the activation of immune cells and the inflammatory cytokine burst. These data highlight the important role of a balanced NPY and ANS interplay in maintenance of the lung immune response in injury.

### Effects of hyperoxia and NPY deficiency on extracellular matrix (ECM) remodeling in neonatal mice

**Fig. 6:**
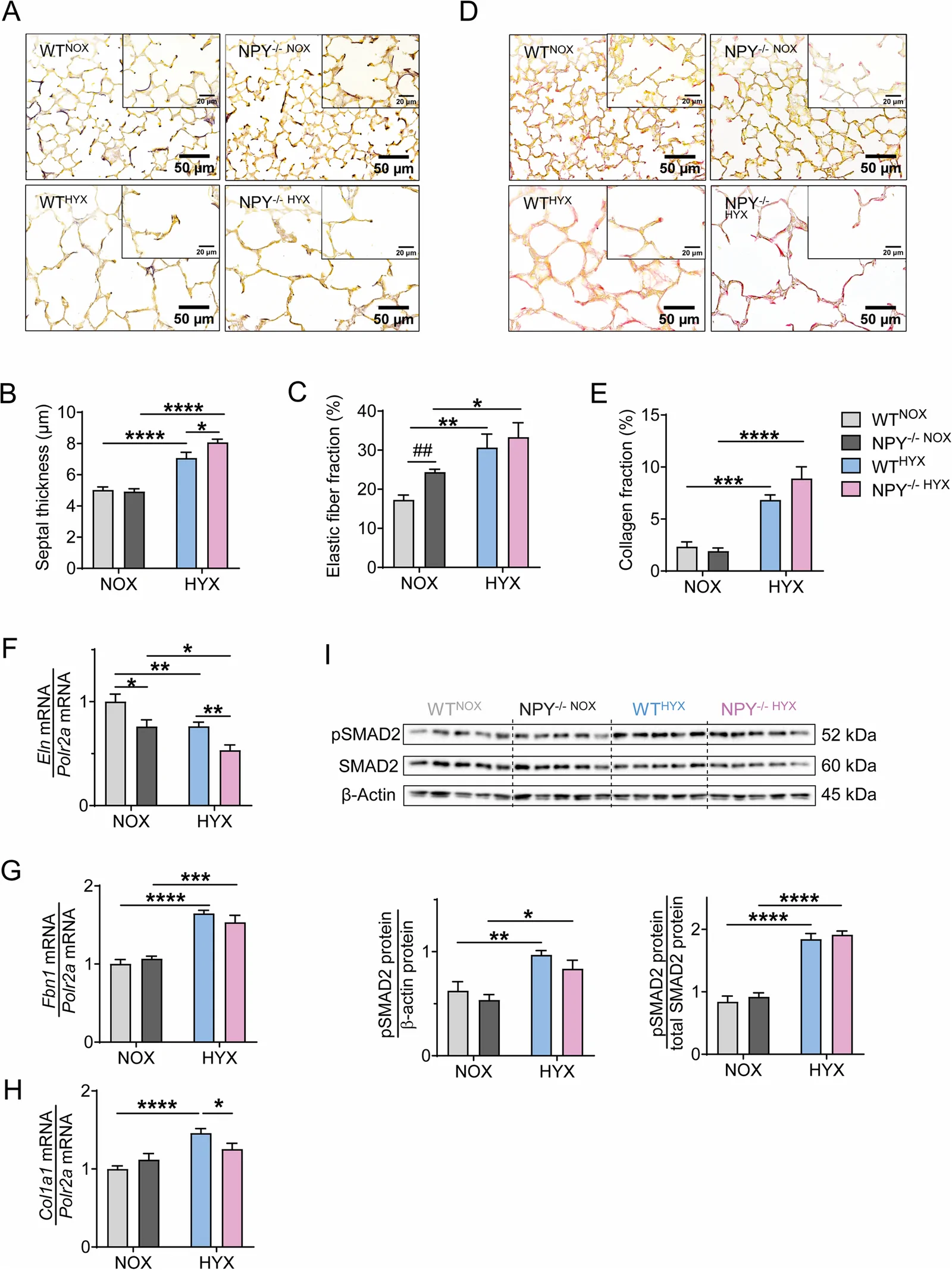
**A**: Representative images showing Hart staining (elastic fibers) in lungs of newborn wildtype (WT) and NPY knockout mice (NPY-/-) exposed to 21% O_2_ (normoxia, NOX) or 85% O_2_ (hyperoxia, HYX) from birth (postnatal day 0, P0) to P14 (A). **B**: Measurement of septal thickness in lungs of WT_NOX_, NPY^-/- NOX^, WT^HYX^, and NPY^-/- HYX^. **C**: Assessment of elastic fiber density relative to lung tissue in %. **D**: Representative images showing Sirius-Red staining in lungs at P14. **E**: Analysis of collagen content relative to lung tissue in % (n=4-6 animals per group and 4-8 lung sections per animal were analyzed). **F-H**: Gene expression of elastin (*Eln*) (F), Fibrillin 1 (*Fbn1*) (G), collagen1α1 (*Col1a1*) (H) using quantitative RT-PCR; *Polr2a* served as housekeeping gene (n=6-12 animals per group). **I**: Immunoblot showing phosphorylated (p)SMAD2 and total SMAD2 in total lung homogenate at P14; pSMAD2 was related to total SMAD2 or to β-Actin that served as loading control (n=6-12 animals per group). The densitometric summary data are shown under the respective immunoblot. All data are presented as mean ± SEM. Statistical significance was assessed using Two-way ANOVA with Bonferroni post-test: *p<0.05, **p<0.01, ***p<0.001, ****p< 0.0001

Since inflammation drives extracellular matrix (ECM) remodeling, we next analyzed structural and molecular alterations in the ECM of WT and NPY_−/−_ pups under normoxic and hyperoxic conditions. Interestingly, hyperoxia caused a significant thickening of alveolar septa in both WT and NPY^−/−^ pups. However, this effect was more pronounced in NPY^−/− HYX^ with a significantly greater septal thickness than WT^HYX^. Since hyperoxia is known to disrupt elastic fiber assembly, leading to abnormal brush-like fiber deposition in the septae [24], we next focused on two key components of the ECM and investigated elastic fibers and collagen content. Hart’s staining suggests disruption of elastic fiber assembly with a loss at the tip of secondary crests and brush-like structures in the alveolar septae. Moreover, the quantitative analysis revealed a significant increase in the elastic fiber fraction in both WT and NPY^−/−^ pups following hyperoxia. Notably, this increase was less pronounced in NPY^−/−^ mice, that already exhibited a significantly elevated baseline elastic fiber ratio under normoxic conditions compared to WT^NOX^. Similarly, hyperoxia led to increased collagen fiber deposition relative to total lung tissue area in WT pups. However, this effect was by trend stronger in NPY^−/− HYX^ compared to WT^HYX^, possibly contributing in part to more thickening of the alveolar septae in NPY^−/− HYX^ compared to WT^HYX^ (Fig. 6A–E). 

Next, we assessed key lung matrix genes, including elastin (*Eln*), fibrillin-1 (*Fbn1*), and collagen 1a1 (*Col1a1*) in total lung homogenates. *Eln* expression was significantly downregulated by WT^HYX^ compared to WT^NOX^. Notably, NPY^−/−^ mice exhibited markedly reduced *Eln* expression under both normoxic and hyperoxic conditions, with significantly lower levels observed in NPY^⁻/⁻ HYX^ compared to WT^HYX^ (Fig. 6F). In contrast, *Fbn1* expression was significantly upregulated by hyperoxia in both genotypes, with no significant difference between WT and NPY^−/−^ (Fig. 6G). Finally, hyperoxia also significantly induced the expression of *Col1α1* in WT^HYX^, and that effect was attenuated in NPY^−/−^ mice. In detail, NPY^−/− HYX^ were protected from the increased *Col1a1* expression compared to both NPY^−/− NOX^ and WT^HYX^ (Fig. 6H), possibly suggesting that assembly and metabolism of collagen, but not gene expression contribute to the slightly increased collagen fiber content in NPY^−/− HYX^.

Since TGF beta signaling is central in fibrotic processes, we examined the activation of its downstream effector SMAD2. First, hyperoxia caused a significant SMAD2 phosphorylation relative to total SMAD2 and the loading control (Fig. 6I). However, no differences in SMAD2 activation were detected between WT and NPY^−/−^ mice in response to hyperoxia. In conclusion, hyperoxia markedly disrupted lung matrix remodeling, leading to increased elastic and collagen fiber content, along with dysregulated assembly and spatial distribution of elastic fibers. Although NPY^−/−^ mice appear to favor fibrotic remodeling of alveolar septa independent of altered activation of SMAD2 signaling, an improved spatial organization of elastic fibers was partially observed.

## Discussion

The present study investigates the functional role of NPY as a sympathetic co-neurotransmitter and immunomodulator in the pathogenesis of hyperoxia-based BPD. In contrast to our initial hypothesis, our findings demonstrate that loss of NPY aggravates hyperoxia-induced arrest of alveolarization, whereas vascular muscularization and, in part, capillary formation appear to be preserved. This dual effect of NPY under hyperoxic conditions in the neonatal lung may be attributable to its role in the fine-tuning of immune responses and the inflammatory microenvironment. Our data further indicate that, although the recruitment of CD68⁺ macrophages to the lung is reduced in NPY^−/−^ mice, the expression of pro-inflammatory cytokines, such as IL-6 and IL-1β, is increased within the local microenvironment. Taken together, these findings suggest a differentially regulated, compartment-specific role of NPY in hyperoxia-based BPD.

The contribution of NPY to normal and aberrant lung development as well as disease progression, particularly in the context of BPD, is poorly understood. In contrast, an increasing body of evidence points towards the emerging role of the ANS in maintenance of lung tissue homeostasis and in the pathogenesis of CLD [25, 26]. NPY signaling contributes to adult lung diseases, including asthma [15, 17, 27], chronic obstructive pulmonary disease (COPD) [28], and pulmonary fibrosis [29]. Notably, clinical studies have also reported enhanced NPYergic innervation in the pulmonary arterial wall in the lungs of patients with PH [11], linking NPY signaling and altered sympathetic activity to lung disease pathology. Here, we now show a significant upregulation of NPY gene expression in neonatal lungs exposed to hyperoxia, suggesting a potential role for NPY in BPD pathogenesis. In addition, the prolonged postnatal exposure to high oxygen concentrations increased neuronal markers, including NeuN, a nuclear marker of postmitotic neuronal cells [30], and PGP9.5, a marker for pulmonary neuroendocrine cells (PNECs) [31]. This was accompanied with increased sympathetic signaling, as evidenced by increased TH expression. Previous studies confirmed a co-localization of NPY with TH, an indicator of the SNS, in the developing rat lung [32]. However, growing evidence suggests a non-neuronal origin of NPY, raising the possibility that other cell types including immune cells may contribute to its production [33, 34]. Indeed, NPY is expressed by activated macrophage-like cells as well as implicated in regulating cytokine production and cellular activities of immune cells in asthma [27]. These results support the notion that NPY is not only produced by the SNS and acts as a sympathetic effector, but also that NPY is expressed independently of the SNS. Indeed, while the SNS acts via NPY on organs and cells, such as immune cells, NPY can also act on the SNS and modulate its function, indicating a bi-directional role of NPY [35, 36].

The hyperoxia-based model of BPD is characterized by impaired alveolar and vascular formation, along with lung matrix remodeling and inflammation [37]. Since the ANS and NPY regulate the immune response and the processes of tissue injury, we tested whether NPY^−/−^ mice are protected from hyperoxia-induced lung injury. Interestingly, our findings indicate a compartment-specific function of NPY. On the one hand, NPY deficiency aggravates the arrest of alveolarization with increased mean linear intercept and average alveolar size. These findings suggest that the elevated abundance of NPY might serve as a protective response to hyperoxia to enable normal alveolarization. In line with our observations, NPY appears to protect against the development and progression of emphysematous changes in the lungs [38]. Similarly, NPY^−/− HYX^ were not protected from reduced number on pre-capillary vessels (20–100 μm), whereas capillary formation (< 20 μm) was partly preserved when compared to WT^HYX^. This discrepancy may reflect distinct underlying mechanisms such as disrupted inflammation and impaired mural cell recruitment in NPY^−/− HYX^. For example, the absence of NPY’s pro-migratory effects may limit recruitment of mural and endothelial precursor cells required for the formation and stabilization of pre-capillary vessels [39, 40]. The mild preservation of capillaries may instead reflect immune microenvironment changes in the alveoli that favor angiogenesis. In line with these findings, NPY deficiency attenuated lung vascular muscularization in hyperoxia-exposed neonatal mice through reducing proliferation of vascular SMCs, indicating a pro-proliferative function. While this is beneficial for alveolar formation under hyperoxia, it also induces vascular remodeling. In support of this, animal models of PH exhibited an upregulated expression of NPY and its Y1-receptor, facilitating vasoconstriction and stimulating the proliferation of pulmonary arterial SMCs, effects that were blocked by Y1-receptor antagonist, confirming the role of NPY in promoting vascular muscularization [11].

Inflammation and alveolar macrophages have been shown to be involved in arrested alveolarization during hyperoxic conditions [41, 42]. In our study, NPY deficiency protected from a hyperoxia-induced increased number of CD68^+^ macrophages in the lung, suggesting that NPY is chemoattractant and vital in the recruitment of macrophages to the lung under hyperoxic conditions. In fact, NPY attracts monocytes and macrophages in pathological conditions such as inflammation and injury [43, 44]. Interestingly, NPY also exerts pleiotropic, and partially opposing effects on different immune cell types, depending on the physiological and pathological contexts [19]. Here, despite the reduced macrophage recruitment and CD68⁺ cell abundance in the absence of NPY, the expression of key pro-inflammatory cytokines, such as *Il*6 and *Il1b*, were significantly elevated in response to hyperoxia in the lungs of NPY^−/−^ mice with a 20-fold and a 3-fold increase for *Il*6 and *Il1b*, respectively, compared to WT. These results may reflect the complexity and the heterogeneity of the immune responses after the acute injury by hyperoxia as lung macrophages comprise both tissue-resident alveolar macrophages (TR-AM) and recruited bone-marrow-derived macrophages (BMDM) that react differently during this acute injury [45]. Thus, in the absence of NPY, the reduced CD68⁺ cell abundance may not indicate reduced inflammatory activity but rather altered activation state of the different macrophage subsets. These finding indicate a dual and context-dependent immunomodulatory function of NPY in neonatal mouse lungs exposed to hyperoxia: on one hand, NPY is chemoattractant and triggers the macrophage recruitment to the lungs; on the other hand, NPY appears to mitigate the inflammatory response to hyperoxia and acts in part anti-inflammatory. This duality reflects an immunomodulatory versus pro-migratory function of NPY with regulation of lung residential macrophages and recruitment of macrophages, respectively. Of note, pro-inflammatory cytokines such as IL-6 and IL-1β can also be released by other cell types, including epithelial and endothelial cells, as well as other immune populations [46, 47]. Thus, the increased cytokine expression observed in total lung homogenates might reflect broader activation of lung cell populations other than macrophages. Furthermore, hyperoxia activates STAT3 phosphorylation in neonatal lungs, a central inflammatory signaling effector of numerous inflammatory cytokines, notable amongst those IL-6 [5]. However, in our study, despite the elevated *Il6* expression, STAT3 phosphorylation, was reduced in lungs of NPY^HYX^ compared to WT^HYX^, suggesting that NPY regulates and enhances STAT3 activation. Thus, this reduction in STAT3 phosphorylation indicates that the absence of NPY might attenuate inflammatory cellular response to hyperoxia. Moreover, various reports indicate an interplay between NPY and STAT3, with STAT3 being activated by NPY and, conversely, NPY expression being inhibited by STAT3 [48, 49]. Further studies are required to understand the origin of elevated cytokine expression and the cell-specific activation of STAT3 in the lungs of NPY^−/−^ mice.

Oxidative stress-triggered inflammation has a direct implication in disrupting and remodeling the extracellular matrix (ECM) [20, 50, 51]. In BPD, where inflammation is persistent, this dysregulated remodeling results in an excessive ECM components’ deposition, fibrosis and eventually further compromising lung function. In line with prior studies [5, 20], we confirm a pronounced increase in alveolar septal thickness in WT mice under hyperoxia, which is further aggravated by NPY deficiency. The increased alveolar septal thickness observed in NPY^−/−^ mice under hyperoxia may be explained by altered lung matrix composition, including a slight increase in collagen fraction and changes in elastic fiber composition, with partially less disrupted elastic fibers. Given the established role of the TGF-β/SMAD2 pathway in fibrosis and hyperoxia-induced neonatal lung injury [52, 53], we assessed SMAD2 phosphorylation. While we confirmed activation of SMAD2 by hyperoxia, we did not observe any additional effect in NPY^−/−^ mice. We therefore speculate that the observed matrix alterations may instead be driven by a more pro-inflammatory microenvironment, characterized by increased *Il6* and *Il1b* expression in NPY^−/− HYX^. Moreover, NPY has a modulatory function on myofibroblasts during lung development by promoting survival and migration that could favor alveolar growth [32]. For example, endogenous NPY1-36 and PYY1-36 can adversely affect cardiac structure/function by activating cardiac fibroblasts [54]. Here, we show that NPY deficiency partially promotes collagen accumulation in developing lungs exposed to hyperoxia and attenuates the activation of elastic fiber formation. In line with our findings, Taniguchi et al., 2022 reported the protective effects of NPY against elastase-induced pulmonary emphysema [38]. Moreover, recent data demonstrate that NPY could play a protective role against pulmonary fibrosis by suppressing IL-1β release in adult mice [16].

At this point we want to emphasize that our murine model of BPD is based on 85% oxygen from birth to P14. This represents a severe injury to the lung with reduced alveolar and vascular formation and marked remodeling of the lung matrix towards fibrotic changes [20]. This model thus corresponds to severe BPD in premature infants and should be interpreted as such. Studies show that different phenotype of hyperoxia-based BPD can be induced depending on the oxygen concentration. At 40% and 60% oxygen, lung growth is inhibited, but matrix remodeling is mild [55]. In future studies, it is therefore important to investigate the role of NPY and the SNS (i) in different severity of BPD as well as the (ii) cell-specific and (iii) age-dependent role of NPY, particularly in lung development versus the mature lung.

## Conclusion

**Fig. 7:**
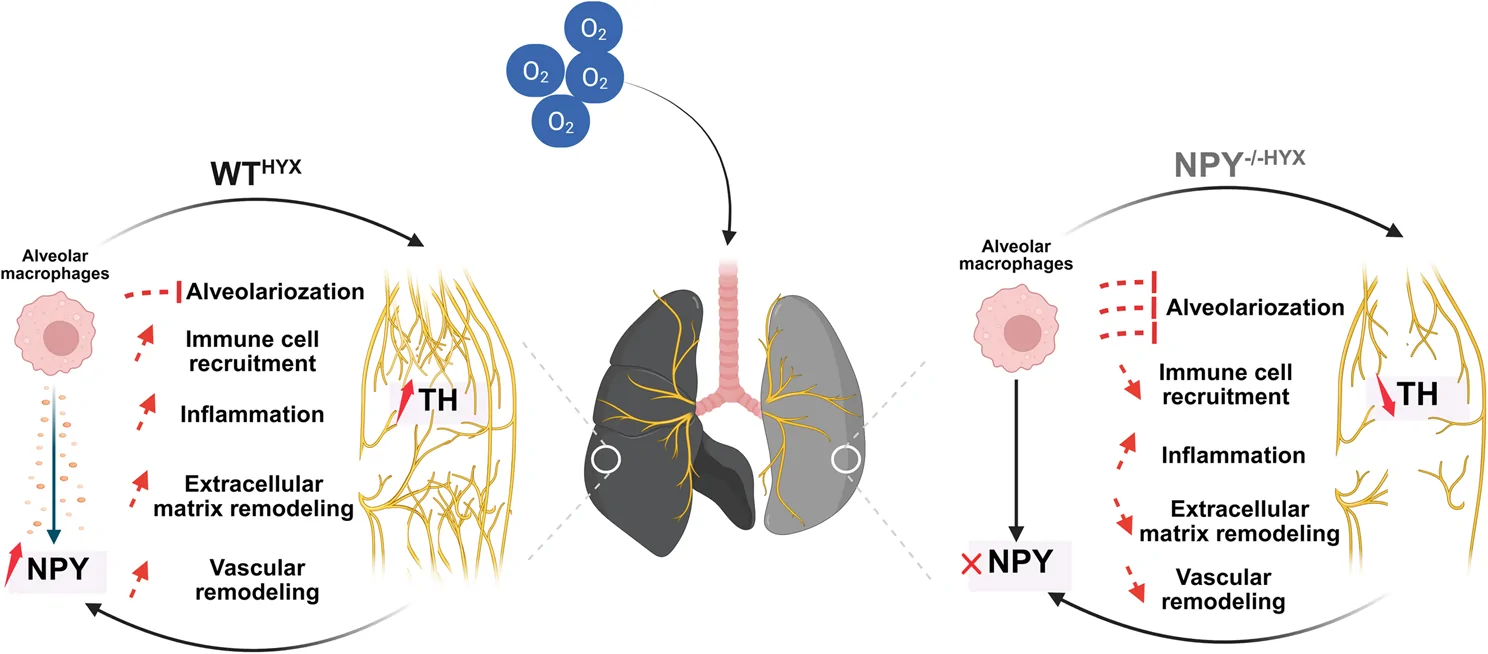
**Proposed working model**. Scheme that illustrates the functional role of NPY and its interaction with the sympathetic nervous system (SNS) in hyperoxia-induced neonatal lung injury, a model of bronchopulmonary dysplasia (BPD). Our data reveal the complex and dual functions of NPY in aberrant alveolarization. Loss of NPY attenuates immune cell recruitment to the lung but, conversely, exacerbates alveolar growth arrest and enhances lung intrinsic inflammation. Created in BioRender. Aejandre Alcazar, M. (2026) https://BioRender.com/redldah

Our study shows a possible functional role of NPY and its interplay with the SNS in a hyperoxia-based model of BPD. The results highlight the complex and often dual and opposing function of NPY in aberrant alveolarization. While the loss of NPY in part protects against vascular muscularization as well as capillarization, it aggravates the arrest of alveolar formation. These structural changes may be mediated by an altered lung immune response, in which NPY deficiency and the associated reduction in SNS activity limit immune cell recruitment to the lung while promoting local cytokine expression (Fig. 7). The great importance of NPY and the SNS in alveolarization and experimental BPD is highlighted in this study and underscores the need for future studies to address NPY signaling pathways and receptors as well as its cell-specific functions in the developing lung.

## Supplementary Information

Supplementary Material 1.

Supplementary Material 2.

Supplementary Material 3.

## Data Availability

We re-analyzed a previously published, publicly available scRNA-seq dataset from newborn mice (Creative Commons Attribution 4.0 international license ( [https://creativecommons.org/licenses/by/4.0/](https:/creativecommons.org/licenses/by/4.0) ). ScRNA sequencing data used in this study, including fastq sequencing files, gene expression matrices, and cell metadata are deposited in the NCBI’s Gene Expression Omnibus (GEO) database (accession code GSE151974).
