# Supplementary material for: The autonomic nervous system-lung interface in experimental BPD: NPY modulates immune response, alveolar growth and vascular muscularizationin neonatal mice exposed to oxidative stress
Source: Respir Res. 2026 Jul 1;27:284. doi: 10.1186/s12931-026-03773-5 (PMC13366976; doi:10.1186/s12931-026-03773-5)

Supplementary material – original western blots
Nies O, Zeitouny C, et al.,

Figure 1

NeuN

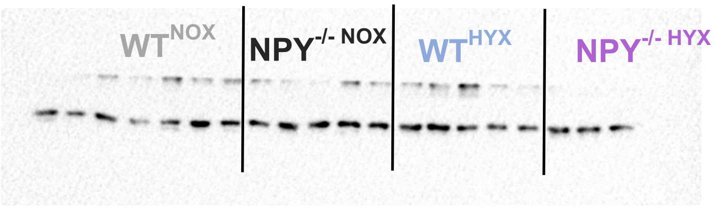

44, 48 kDa

PGP.9

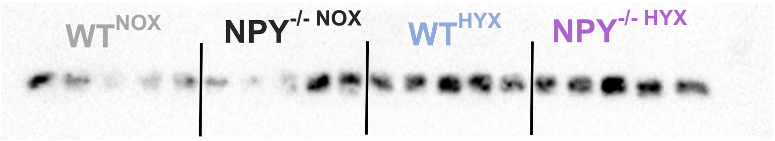

25 kDa

TH

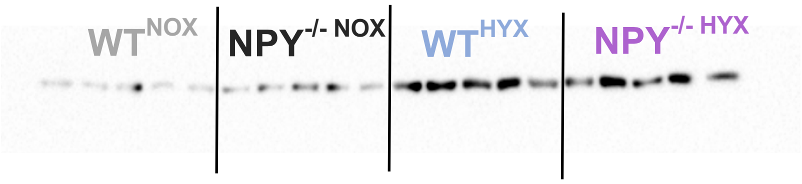

59 kDa

Beta-Actin

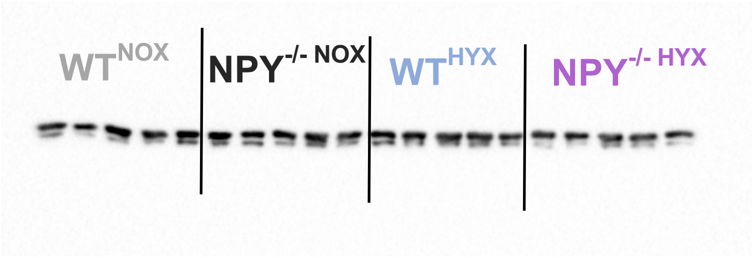

45 kDa

Supplementary material – original western blots
Nies O, Zeitouny C, et al.,

Figure 3

VE-Cdh

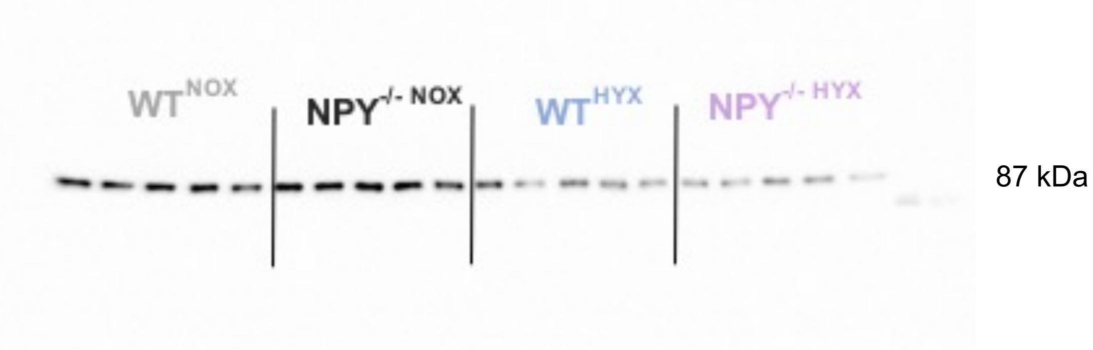

Beta-Actin (VE-Cdh)

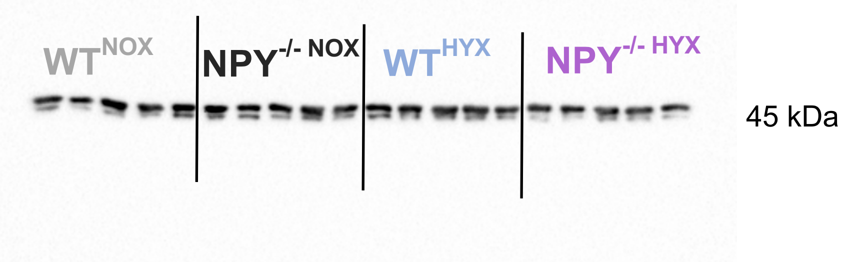

PECAM (CD31)

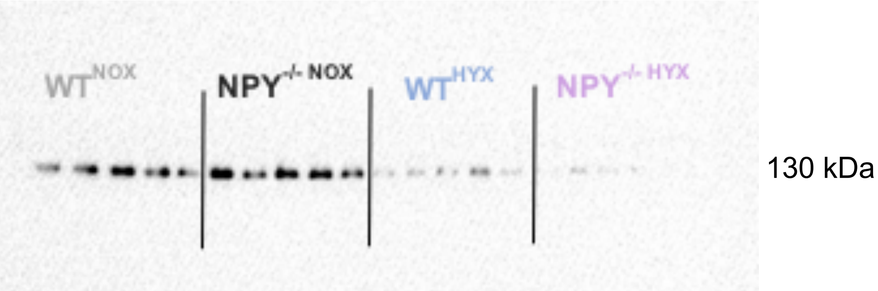

Beta-Actin (PECAM, CD31)

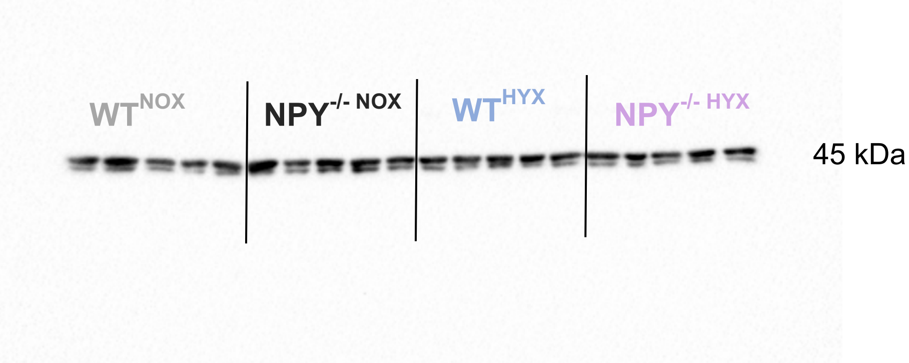

Supplementary material – original western blots
Nies O, Zeitouny C, et al.,

Figure 4

Alpha-SMA

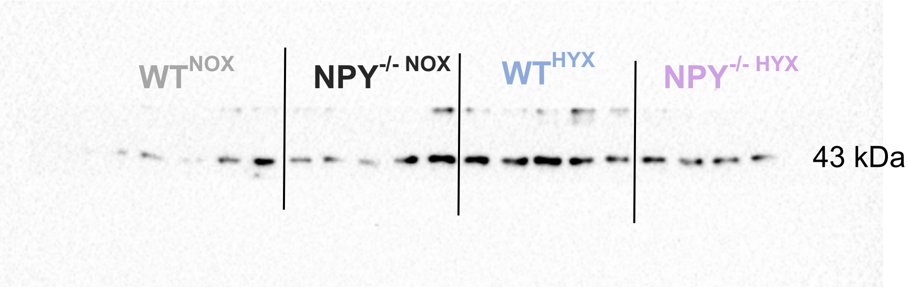

43 kDa

Beta-Actin (alpha-SMA)

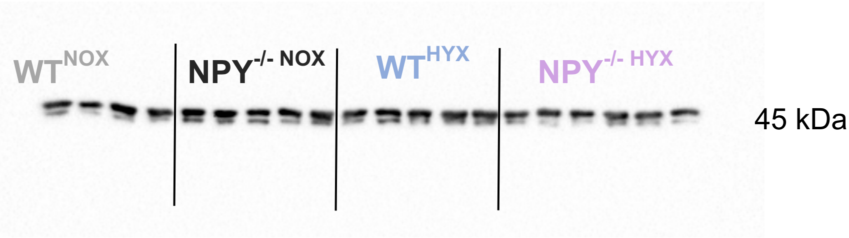

45 kDa

Figure 5

Phospho STAT3 (pSTAT3)

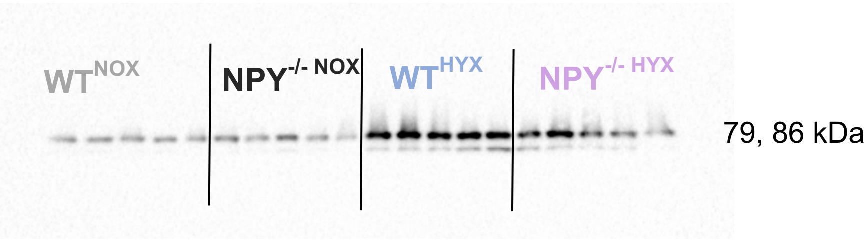

79, 86 kDa

Total STAT3

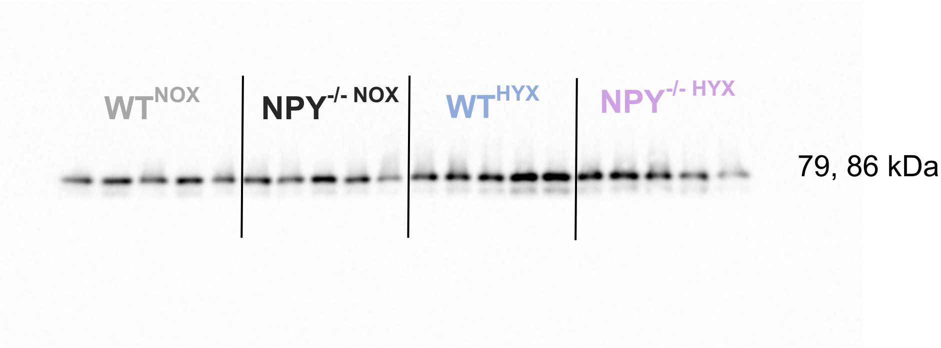

79, 86 kDa

Beta-Actin (pSTAT3 and total STAT3)

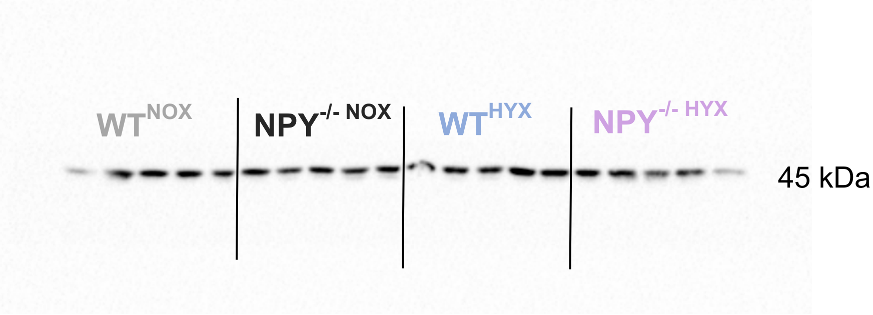

Phospho P42/44 (pP42/44)

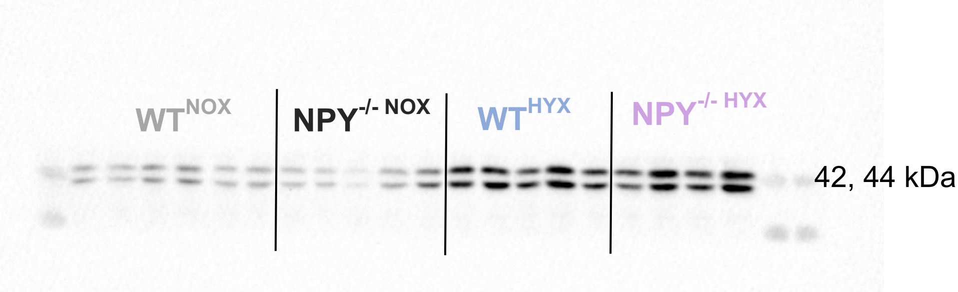

Total P42/44 total

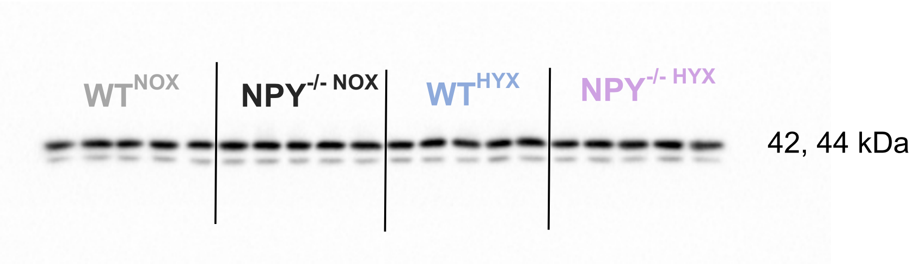

Beta-Actin (pP42/44 and total P42/44)

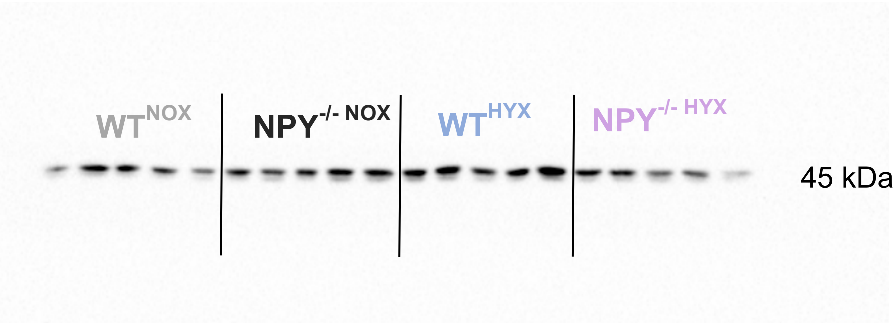

Figure 6

Phospho SMAD2 (pSMAD2)

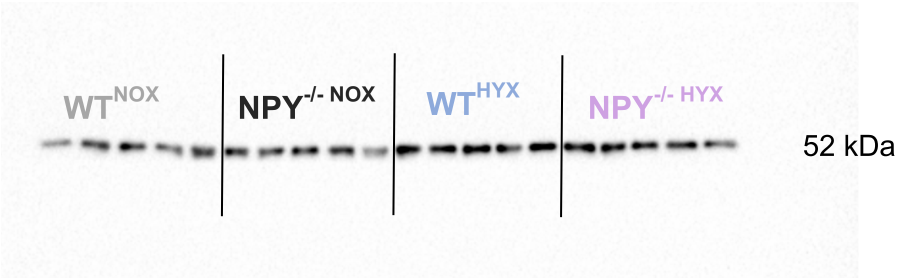

Total SMAD2

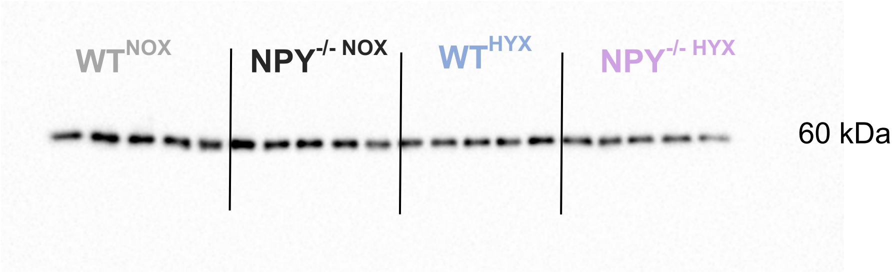

Beta-Actin (pSMAD2 and total SMAD2)

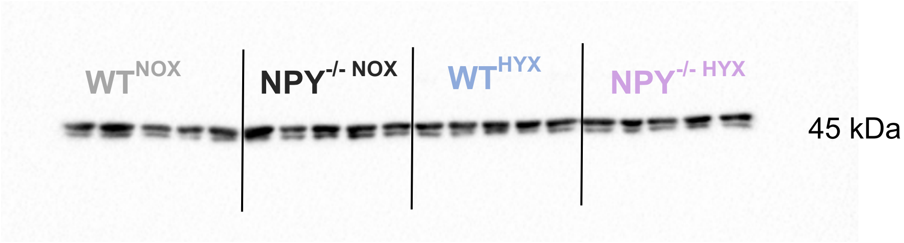

Supplement: Supplementary file 2 — Supplementary Material 2. [file 12931_2026_3773_MOESM2_ESM.pdf]
