# Supplementary material for: The autonomic nervous system-lung interface in experimental BPD: NPY modulates immune response, alveolar growth and vascular muscularizationin neonatal mice exposed to oxidative stress
Source: Respir Res. 2026 Jul 1;27:284. doi: 10.1186/s12931-026-03773-5 (PMC13366976; doi:10.1186/s12931-026-03773-5)
Supplement: Supplementary file 3 — Supplementary Material 3. [file 12931_2026_3773_MOESM3_ESM.docx]

**Detailed Materials and Methods**

**Animal studies**

Animal studies were conducted in compliance with German regulations and legal requirements, approved by the local government authorities (LANUV, NRW, Germany ; AZ8.87-51.04. 2010.A372; 84-02.04. 2015.A120; AZ8.87-51.05.20.11.013). On the day of birth (postnatal day 0, P0), female and male wildtype (WT) C57BL/6N and NPY- Knockout (NPY^-/-^) B6.129S-NPYtm1Rpa/J pups were randomly assigned to experimental groups and exposed to 85% O2 (hyperoxia, HYX) in a Biospherix chamber and ProOx 110 Cytocentric® High Infusion Rate O2 Controller (Biospherix, Parish, NY, USA). Control pups were maintained in room-air (normoxia, NOX, 21% O2) as previously described [1].

All mice were euthanized at P14 as previously described [1]. Anesthesia was administered via intraperitoneal injection of Xylazine (10 mg/kg body weight) and Ketamine (100 mg/kg body weight). The right lungs were excised, snap-frozen, and stored at -80°C for molecular analyses. The left lungs were inflated by pressure fixation at a constant pressure of 20 cm H2O using fixative agent [4% (mass/volume) of paraformaldehyde (PFA)] for 15 min before being excised and subsequently fixed in 4% PFA in phosphate-buffered saline (PBS) (4% Histofix, Carl Roth, Germany, #P087) overnight before being fixed in paraffin for histological analyses.

**Tissue assays**

**Protein extraction and Western Blot**

Protein extraction followed by immunoblotting was carried out as described previously [1]. Frozen lungs were homogenized and lyzed in CHAPS buffer (Calbiochem, USA, #220201) and Halt™ protease/phosphatase inhibitor solution (Thermo Fisher Scientific, USA, #78442). Cell membranes were disrupted via sonication using the SONOPLUS HD 2070 ultrasonic homogenizer (Ultraschall-Homogenisator + GM2070 Stab SH 70G; Bandelin, Germany, #517.00001672.012). The homogenates were incubated on ice for 1 hour with intermittent mixing, followed by centrifugation at 15,000 rpm at 4°C. The supernatant was collected for further analysis. Protein concentrations were determined using the BCA Protein Assay Kit (Thermo Fisher Scientific, USA, #23225) according to the manufacturer’s instructions.

For protein separation, 20 µg of protein per sample was prepared for SDS-PAGE by incubation with reducing sample loading buffer and subsequently loaded into the wells of a 10% acrylamide gel. Electrophoresis was performed at 120 V and 120 mA. Proteins were then transferred onto a nitrocellulose membrane (Carl Roth, Germany, #4675.1) using a constant current of 1.3 mA/cm². To prevent non-specific binding, membranes were blocked with 5% milk powder and 2% bovine serum albumin (BSA) in Tris-buffered saline with Tween-20 (TBS-T). The membranes were incubated overnight at 4°C with the following primary antibodies: mouse anti- β-Actin (Cell signaling Technology, USA, #3700, 1:10.000 in 5% milk powder in TBS-T), rat anti- PECAM (CD31; Dianova, DIA310, 1:5000 in 5% BSA in TBS-T), mouse anti-NeuN (abcam, UK, ab104224, 1:1000 in 5% milk powder in TBS-T), rabbit anti- P42/44 (ERK) (Cell signaling Technology, USA, #9102, 1:2000 in 5% BSA in TBS-T), rabbit anti- phosphor P42/44 (pERK) (Cell signaling Technology, USA, #4370, 1:2000 in 5% BSA in TBS-T), rabbit anti- PGP9.5 (abcam, UK, ab27053 , 1:1000 in 5% BSA in TBS-T), mouse anti-ACTA2 (Santa Cruz, sc53142, 1:1000 in 5% milk powder in TBS-T), rabbit anti- SMAD2 XP (Cell signaling Technology, USA, #5339, 1:1000 in 5% BSA in TBS-T), rabbit anti- phosphor SMAD2 (S467) (abcam, UK, ab280888, 1:1000 in 5% BSA in TBS-T), mouse anti-STAT3 (Cell signaling Technology, USA, #9139, 1:3000 in 5% milk powder in TBS-T), rabbit anti-pSTAT3 (Tyr705) XP (Cell signaling Technology, USA, #9145, 1:1000 in 5% BSA in TBS-T), rabbit anti-TH (abcam, UK, ab112, 1:500 in 5% BSA in TBS-T), and rabbit anti-VE-Cadherin (abcam, UK, ab33168, 1:1000 in 5% BSA in TBS-T). Following primary antibody, membranes were incubated with horseradish peroxidase (HRP)-conjugated secondary antibodies, including anti-rabbit (Cell Signaling Technology, USA, #7074S, anti-mouse (Cell Signaling Technology, USA, #7076,), and anti-rat (Cell Signaling Technology, USA, #7077) at room temperature for 1 hour. Protein detection was carried out using a chemiluminescent substrate (Amersham ECL Prime, Cytiva, Germany, #RPN2232), applied for 1 minute. Chemiluminescent signals were captured using the Molecular Imager ChemiDoc XRS+ system (Bio-Rad Laboratories, USA, #721BRO2933). Densitometric analysis was performed using Image Lab software 5.2.1 (Bio-Rad Laboratories, USA).

**RNA extraction, cDNA synthesis, and quantitative reverse transcriptase polymerase chain reaction PCR (qRT-PCR)**

Frozen lung samples were lyzed using TRI- Reagent™ (Sigma-Aldrich, USA, #93289). RNA was separated using chloroform, then precipitated with isopropanol. After ethanol washing, RNA was rehydrated in diethyl pyrocarbonate-treated RNase-free water (Sigma-Aldrich, USA, #D5758) and quantified using the Infinite M200 Pro NanoQuant spectrophotometer (Tecan Group, Switzerland, #1010003100). For cDNA synthesis, 1 µg of RNA was treated with an RNase-free DNase kit (Promega, USA, #M6101) according to manufacturer´s instructions to remove genomic DNA. Random and oligo Primers (Roche, Germany, #11034731001; Eurofins Genomic, Germany, #16-T) were added, followed by heating at 70°C to disrupt secondary RNA structures. M-MLV Reverse Transcriptase (Promega, USA, #M1705), its buffer (Promega, USA, #M5313), RNase-free inhibitor solution (Promega, USA, #N2515) and dNTPs (Fermentas, Lithuania, #R0193) were subsequently added. Reverse transcription was carried out at 37°C for one hour, followed by cooling on ice for one minute. Quantitative RT-PCR was performed using reaction solutions that were prepared in FrameStar® 96-well semi-skirted PCR plates (4titude Ltd. Germany, #4ti-0770/C), diluted with HLPC-water (Merck, Germany, #1153331000). Master mixes for Taqman and SYBR assays were added accordingly (Invitrogen™ Platinum™ Quantitative PCR Supermix-UDG w/ROX, Invitrogen, USA, #11743500 for Taqman-qPCR, or GoTaq® qPCR Master Mix, Promega, USA, #A6002 for SYBR-qPCR). The PCR amplification was performed following the manufacturer´s recommended settings of 40 cycles. Quantitative analysis was conducted using the 7500 Real-Time PCR System (Applied Biosystems, USA), as previously described [2]. Primer sequences were designed using Prime-BLAST software (National Center for Biotechnology Information, USA). The sequences used are depicted in supplementary *Table 1*. Changes in gene expression were determined using the ∆∆Ct-method with *Polr2a* serving as reference gene. Results were expressed as fold-induction.

***Supplementary Table 1. List of*** *Primers used for qRT-PCR. *SYBR-Green Primers*

| **Gene** | **Species** | **Primer** | **Sequence** |
| --- | --- | --- | --- |
| *Apelin (*Apln*) | mouse | for | CCCCTTTTAAGTCCTTTGGCATCT |
|  |  | rev | CTCGCTAAAAAGTCCCGAAAGTAT |
| CD31 (*Pecam1*) | mouse | for | AAAGCCAAGGCCAAACAGAA |
|  |  | rev | CCAGAAACATCATCATAACCGTAATG |
|  |  | probe | AGATGTCCAGGCCAGCTGCTCCACTT |
| VE-Cadherin (*Cdh5*) | mouse | for | TGGCCAAAGACCCTGACAAG |
|  |  | rev | TCGGAAGAATTGGCCTCTGT |
|  |  | probe | CTCAGCGCAGCATCGGGTACTCCAT |
| *Collagen 1alpha 1 (Col1a1) | mouse | for | GCAGTGCTGTTGCGATCTTG |
|  |  | rev | CAGAGGGACAGAGCACAGCTT |
| *Elastin (*Eln*) | mouse | for | CTACGGACTGCCCTATACCAATG |
|  |  | rev | CACCATACTTGGCTGCTTTAGCT |
| *Fibrillin1 (*Fbn1*) | mouse | for | GGTCAATGCAACGATCGAAA |
|  |  | rev | AGTGTGACAAAGGCAGTAGAAGCTT |
| IL-1 beta (*Il1b*) | mouse | for | TGACAGTGATGAGAATGACCTGTTC |
|  |  | rev | GGACAGCCCAGGTCAAAGG |
|  |  | probe | ACCCCAAAAGATGAAGGGCTGCTTCC |
| IL-6 (*Il6*) | mouse | for | ACAAGTCGGAGGCTTAATTACACAT |
|  |  | rev | AATCAGAATTGCCATTGCACAA |
|  |  | probe | TCTTTTCTCATTTCCACGATTTCCCAGAGAA |
| IL-10 (*Il10*) | mouse | for | GGCGCTGTCATCGATTTCTC |
|  |  | rev | CACCTTGGTCTTGGAGCTTATTAAA |
|  |  | probe | CCTGTGAAAATAAGAGCAAG |
| *PDGF-receptor ⍺ | mouse | for | GGTCGAAGGTACAGCTTATGGATT |
| (*Pdgfra*) |  | rev | CCCCAGGTGAGTCATTATCTTCA |
| RNA polymerase II Subunit A (*Polr2a*) | mouse | for | TTTGGCGATGATTTGAATTGTATC |
|  |  | rev | ACCTCTTCCTCCTCTTGCATCTT |
|  |  | probe | CAGAGAAGCTGGTCCTTCGAATCCGC |
| *Npy1r* | mouse | for | CCACCTGCAACCACAATCTG |
|  |  | rev | GGTTGACGCAGGTGGAGATC |
|  |  | probe | TTTCTGCTCTGTCACCTCACCGCCA |
| *Npy2r* | mouse | for | GCGGTACAAGTGTCCACAATAACT |
|  |  | rev | GATCTTGCTCTCCAGGTGGTAGA |
|  |  | probe | ACAGTCATTGCTCTGGACCGCCATC |
| **Polr2a* | mouse | for | CTA AGG GGC AGC CAA AGA AAC |
|  |  | rev | CCA TTC AGC ATA CAA CTC TAG GC |
| Supressor of cytokine signaling 3 (*Socs3*) | mouse | for | CCACCCTCCAGCATCTTTGT |
|  |  | rev | TCCAGGAACTCCCGAATGG |
|  |  | probe | ACTGTCAACGGCCACCTGGACTCCT |
| Tumor necrosis factor alpha (*Tnfa*) | mouse | for | GGCTGCCCCGACTACGT |
|  |  | rev | GACTTTCTCCTGGTATGAGATAGCAA |
|  |  | probe | CCTCACCCACACCGTCAGCCG |

**Single cell RNA sequencing (scRNA-seq) data**

To assess the number of Npy+ cells within the alveolar macrophage population, as well as the expression of Npy gene in the developing mouse lungs at single-cell level we re-analyzed a previously published, publicly available scRNA-seq dataset from newborn mice (Creative Commons Attribution 4.0 international license (<https://creativecommons.org/licenses/by/4.0/>) [3]. The whole-lung UMAP (Figure 1F) is a replication of Figure 2A in the original article [3]. Data shown in Figure 1G were exclusively created for this manuscript with the above-mentioned available dataset. Analyses were performed using R studio, R version 4.2.3. Details on proportion of *Npy*^+^ cells in lung myeloid cells are summarized in table 2 (provided as an Excel file). Proportion of *Npy*^+^ cells in alveolar macrophages cluster was analyzed with GraphPad Prim 10.2.1.

ScRNA sequencing data used in this study, including fastq sequencing files, gene expression matrices, and cell metadata were previously published [3] and deposited in the NCBI’s Gene Expression Omnibus (GEO) database (accession code GSE151974).

**Quantitative histomorphometric, immunohistochemical and immunofluorescent analysis**

Histomorphometric analysis of the lung tissue was performed as described previously [1, 4]. PFA fixed, paraffin-embedded lung samples were sectioned into 3 µm thick slices and mounted onto poly-L-lysine-coated glass slides. After treatment with NeoClear (Merck, Germany, #1098435000), sections were rehydrated through descending concentrations of ethanol (100%, 96%, 70%) followed by distilled water. The lung tissue was then stained using hematoxylin (Carl Roth, Germany, #T865.2) and 0.5% eosin (Carl Roth, Germany, #X883.2) in 1% acetic acid solution. Next, slides were dehydrated using ascending concentrations of ethanol, treated with additional Neoclear and mounted for analysis. For each group, histological analysis was performed on four to five slides from five randomly selected animals, up to ten images per slide were included in the analysis. The Mean Linear Intercept (MLI), Radial Alveolar Count (RAC), and Alveolar Septal Thickness (AST) were assessed using light microscopy, slide scanner (Leica SCN400), and Cell D 3.4 Olympus soft image solutions (Olympus, Hamburg, Germany). as previously described [4].

**Elastic fibers staining and quantification**

Paraffine-embedded lung-sections (prepared and rehydrated as described previously) were stained with 10% Resorcine Fuchsine from Weigert (Waldeck Chroma, Germany, #2.00E-30) in 1% hydrochloric acid in 70% ethanol overnight followed by counter staining with 0.5% Tartrazine (ScyTek Laboratories, USA, #TZQ125) in 0.25% acetic acid for 30 minutes. Photomicrographs for quantification were taken using a microscope [Olympus BX43, (Olympus, Shinjuku, Tokyo, Japan)] at 40x magnification using CellSens Dimension software (Olympus, Shinjuku, Tokyo, Japan), collecting up to 10 images per section of 5-6 animals. Elastic fiber density in relation to total lung tissue was determined using Image J software.

**Collagen fibers staining and quantification**

Paraffine-embedded lung-sections were deparaffinized and rehydrated as described above. Slides were then stained with Picrosirius Red Solution (ScyTek Laboratories, USA, #SRS500) and counterstained with 0.2% phosphomolybdic acid solution (Carl Roth, Germany, #4440.1). Subsequently, images were processed, edited, and analyzed to assess the ratio of alveolar collagen fiber density and total lung tissue.

**Immunohistochemistry**

*For CD68 staining*, tissue sections were deparaffinized and rehydrated as previously described. Afterwards, antigen retrieval was performed by boiling slides with 10 mM Citrate buffer pH 6 (Dako, Germany, #S2369) at 99 °C for 25 minutes. Next, the tissue sections were treated with a blocking solution (Sea Block, Thermo Scientific™, Netherlands, #37527) at RT for 1 hour. Slides were incubated overnight with the primary antibody rabbit anti-CD68 (Abcam, UK, ab125212, 1:200) at 4 °C. Next, the slides were washed with PBS and then exposed to the secondary antibody (Histofine® MOUSESTAIN KIT, Nichirei, #414341F, Japan, for rabbit primary antibody) for 1 hour at RT, followed by mounting using Neo-Mount® (Merck, Germany, #109,016). The whole tissue section was scanned with a slide scanner (Leica SCN400 Slide Scanner, Houston, USA). CD68^+^ cells (macrophages) were counted in up to ten fields of view per slide and randomly selected lung sections per animal from 5-7 animals per group were studied. The analysis was carried out at 20x magnification using Image Scope and ImageJ software. To ensure accurate assessment, large vessels, airways, and collapsed alveolar areas were excluded from the analysis. The number of CD68^+^ cells was determined per image (Region of Interest, ROI) at 20x magnification.

*For ACTA2 and vWF staining,* tissue sections were also deparaffinized and rehydrated. Antigen retrieval was carried outin 10 mM Citrate buffer (pH 6) at 99 °C for 20 min. Next, lung sections were treated with an avidin-biotin blocking kit (Biozol, Vec-SP-2001 Vector) for 1 hour. Slides were then incubated with the primary antibody anti-ACTA2 (Abcam, UK, ab125057 1:200) for 1 hour at RT. Slides were then incubated with Streptavidin-HRP (Dako, P0397, 1:300 in 1M TRIS, pH 7.4) for 30 minutes. Afterwards, sections were incubated with DAB substrate solution (ImmunoLogic, VWR, BS04-110) for 10 minutes. A second primary antibody, anti-vWF (Dako, A0082, 1:200), was applied for 30 minutes. Slides were then incubated with AP Polymer (Histofine SimpleStain AP, 414261F) for 30 minutes, followed by PBS washing. Fast Red substrate (Biozol, ZYT-ZUC001-125) was applied for 5 minutes, and slides were rinsed with distilled water. Nuclei were counterstained with hematoxylin for 5 minutes. Slides were then dehydrated through an ascending alcohol series, cleared in Neo-Clear, and mounted with Neo-Mount (Merck, Germany, #109016).

*For the assessment of vascular density*, the number of transversally cut vessels with a diameter of less than 20 µm and a diameter between 20 and 100 µm were assessed and normalized to the lung area within the visceral pleura.

*For measurement of vascular muscularization*, transversally cut vessels with diameter between 20 and 100 µm were divided into three groups, according to their muscular circumference: non-muscularized (< 20%), partially muscularized (20 - 70%), and fully muscularized (> 70%). The proportion of vessels in every group were then normalized to the lung area. Additionally, the Medial Wall Thickness (MWT) of each vessel was assessed by measuring the thickness of the medial wall at 3 defined areas. Then, the average of these measurements was calculated and related to the vessel’s diameter.

**Immunofluorescent staining**

Lung tissues were deparaffinized and rehydrated as described above. To reduce autofluorescence the MaxBlock^TM^ reducing kit (MaxVision Biosciences, USA, #M-BL) was applied. For enhanced nucleic antibody binding, sections were treated with a proteinase K (Fermentas, Lithuania, #EO0491, 1:500) in Tris-EDTA-buffer at 37°C for 15 minutes. Afterwards, antigen retrieval was performed by boiling sections in 10 mM citrate buffer for 25 minutes. To prevent non-specific binding, slides were incubated with SEA BLOCK Blocking Buffer (Thermo Fisher Scientific, USA, #37527). For ki-67 staining, slides were then incubated with a primary Ki-67 antibody (Invitrogen, USA, #14-56-98-82, 1:500), followed by an anti-rat fluorochrome conjugated secondary antibody (abcam, UK, #ab150157, 1:1000). To stain for αSMA (ACTA2), anti-mouse αSMA-Cy3-labeled primary antibody (Sigma-Aldrich, USA, #C6198, 1:200) was used. Cell nuclei were stained with 4’,6-diamidino-2-phenylindole (DAPI) (Sigma-Aldrich, USA, #D9542, 1:1000). Fluorescence imaging was conducted at **40× magnification.** In the **muscular compartment of vessels** (diameter **20 - 100 µm),** the number of **Ki-67/αSMA double-positive cells** was quantified and expressed as a ratio relative to the **total number of αSMA-positive cells.**

*For tyrosine hydroxylase (TH) staining*, paraffine-embedded lung-sections were also deparaffinized and rehydrated, followed by a permeabilization step using 0,1% Triton in PBS. Afterwards, antigen retrieval was performed by boiling sections in 10 mM citrate buffer for 25 minutes, followed by blocking with 5% goat serum. To detect TH, sections were incubated with a rabbit-anti-TH antibody (Abcam, Cambridge, UK, # ab112, 1:200), followed by a goat-anti-rabbit AF488 secondary antibody (Jackson Immunoresearch, 1:200). Cell nuclei were counterstained with DAPI (Sigma-Aldrich, USA, #D9542, 1:1000). TH positive area was quantified and expressed as a ratio relative to the **total number of DAPI cells.**

For TH and NPY staining, paraffine-embedded lung-sections were also deparaffinized and rehydrated, followed by a permeabilization step using 0,1% Triton in PBS. Afterwards, antigen retrieval was performed by boiling sections in 10 mM citrate buffer for 25 minutes, followed by blocking with 5% goat serum. To detect TH and NPY, sections were incubated with a rabbit-anti-TH antibody (Abcam, Cambridge, UK, # ab112, 1:200) and with a rabbit anti-NPY staining (cell signaling, 11976, 1:200) followed by a goat-anti-rabbit AF488 secondary antibody (Jackson Immunoresearch, 1:200) and goat anti-mouse IgG conjugated to Cy3 (Jackson Immunoresearch, 1:200).

For quantification, 4 images per animal were taken using a microscope (Olympus BX43, (Olympus, Shinjuku, Tokyo, Japan) at 20x magnification, followed by analysis with the CellSens Dimension software (Olympus, Shinjuku, Tokyo, Japan). Representative images were taken with a confocal laser scanning microscope (Stellaris 5 LIAchroic, inverse, Leica Microsystems, Wetzlar, Germany) at 20x magnification.

**References**

1. Hirani, D., et al., *Macrophage-derived IL-6 trans-signalling as a novel target in the pathogenesis of bronchopulmonary dysplasia.* Eur Respir J, 2022. **59**(2).

2. Sundstrom, G., et al., *Interactions of zebrafish peptide YYb with the neuropeptide Y-family receptors Y4, Y7, Y8a, and Y8b.* Front Neurosci, 2013. **7**: p. 29.

3. Hurskainen, M., et al., *Single cell transcriptomic analysis of murine lung development on hyperoxia-induced damage.* Nat Commun, 2021. **12**(1): p. 1565.

4. Will, J.P., et al., *Strain-dependent effects on lung structure, matrix remodeling, and Stat3/Smad2 signaling in C57BL/6N and C57BL/6J mice after neonatal hyperoxia.* Am J Physiol Regul Integr Comp Physiol, 2019. **317**(1): p. R169-R181.
